# Anomalous structure transition in undercooled melt regulates polymorphic selection in barium titanate crystallization

**DOI:** 10.1038/s42004-021-00462-w

**Published:** 2021-03-02

**Authors:** Xuan Ge, Qiaodan Hu, Fan Yang, Jun Xu, Yanfeng Han, Pingsheng Lai, Jingyu Qin, Jianguo Li

**Affiliations:** 1 Shanghai Key Laboratory of Materials Laser Processing and Modification, School of Materials Science and Engineering, Shanghai Jiao Tong University, Shanghai, People’s Republic of China; 2 School of Mechanical Engineering, Shanghai Jiao Tong University, Shanghai, People’s Republic of China; 3 School of Materials Science and Engineering, Shandong University, Jinan, People’s Republic of China

**Keywords:** Phase transitions and critical phenomena, Structure of solids and liquids, Molecular dynamics, Solid-state chemistry

## Abstract

The crystallization processes of titanates are central to the fabrication of optical and electrical crystals and glasses, but their rich polymorphism is not fully understood. Here, we show when and how polymorphic selection occurs during the crystallization of barium titanate (BaTiO_3_, BT) using in situ high energy synchrotron X-ray diffraction and ab initio molecular dynamic simulation. An anomalous structure transition is found in molten BT during cooling across the cubic-hexagonal transition temperature, which enables nucleation selection of BT by manipulating the undercooling: a cubic phase is preferred if nucleation is triggered at large undercooling, whereas a hexagonal phase is promoted at small undercooling. We further reveal that the nucleation selection between the cubic and the hexagonal phase is regulated by the intrinsic structure property of the melt, in particular, the degree of polymerization between Ti-O polyhedra. These findings provide an innovative perspective to link the polymorphic crystallization to the non-isomorphic structure transition of the melt beyond the conventional cognition of structural heredity.

## Abstract (web summary)

The crystallization processes of titanates are central to the fabrication of optical and electrical crystals and glasses, but their rich polymorphism is not fully understood. Here, authors identify when and how polymorphic selection takes place during the crystallization of an undercooled barium titanate melt using aerodynamic levitation, in situ time resolved high energy X-ray diffraction and ab initio molecular dynamics simulations.

## Introduction

The crystallization process of titanates from their melt is critical to fabrication of many functional materials such as optical/electrical crystals^1–3^, sprayed coatings^4,5^, ferrous metallurgy^6,7^, and high-refractive optical glass^8,9^. There is a universal and interesting phenomenon that titanates exhibit polymorphic phase selection when they nucleate from undercooled melt. For instance, anatase and rutile phase selection occurs during synthesis of TiO_2_ nanoparticles^10^; hexagonal and cubic phase selection exists in the quenching process of BaTiO_3_ (BT) melt^11,12^. To obtain a desired phase, a certain condition at which precipitation of one phase is preferential to other possible polymorphs is required^13^. However, searching for such appropriate condition can be difficult as the polymorphic selection mechanism of titanates is still unclear and therefore requires an in-depth investigation.

As polymorphic selection is widely observed in a large variety of materials^14–18^, lot of efforts have been devoted to understanding the underlying mechanism(s). Early studies attempt to understand the polymorphic selection from the perspective of free energy following the Ostwald’s step rule^19^, and propose that the difference in entropy/enthalpy of fusion between isomers being the reason for polymorphic selection^20,21^. However, such interpretation from the energy viewpoint encounters difficulty in explaining the complicated polymorphic selection behaviors in atomic scale such as the preferential formation of metastable bcc crystallites in the Lennard-Jones system and random stacking or cross-nucleation of hcp/fcc in the hard-sphere system^22,23^. For complex systems (e.g., metals, polymers, oxides, or solutions), understanding the polymorphic selection mechanism is more challenging as it may be influenced by many factors. Taking aluminum as an example, its nucleation is proceeded with random packing of fcc and hcp crystallites in contrast to the prediction that formation of bcc-like phase is preferred. Such discrepancy is discussed from the strong cohesive interaction in Al^24^. Very recently, An et al. proposed that the stability of isomers also encodes the crystallization pathway^17^. Furthermore, nucleation in complex systems is usually proceeded as multi-step scenarios^16,25,26^, which makes the polymorphic selection process a particularly challenging issue for investigation.

To clarify the polymorphic selection mechanism, the following two critical questions should be addressed: (i) When does polymorphic selection take place? From the viewpoint of stepwise energy^19,27^, it should occur during the nucleation stage, but computational simulations on controlling polymorphism^15,17^ suggest that it may occur during the growth stage. (ii) What is the atomic/molecular-scale structural explanation for polymorphic selection? Recent studies by advanced TEM technique reveal that the crystallization process is not a single channel but a crossover of multiple intermediate states^28,29^, which raises the question whether the structural characteristics of these intermediate states can be the origin for polymorphic selection.

Addressing the above questions relies on in situ tracking of the structural evolution of the melt during the nucleation/crystallization process. For titanates, this can be particularly difficult due to high temperature and highly corrosive melt. Fortunately, the advances of containerless processing and its combination with high-energy X-ray diffraction (HEXRD) in synchrotron radiation facilities provide a useful approach to probe the structure of melt^30–32^. Such technique has been successfully applied to many molten oxides to probe their structural features and to investigate the solidification process^33–39^. Results from previous studies have illuminated that the structural evolution of melt has a dramatic impact on the subsequent nucleation behavior. Mechanistic research on the correlation between melt structure and nucleation follows two major mainstreams: (1) Cross-link among structural evolution, macroscopic properties of melt and nucleation. It is proposed that change of the locally order structure of cation–oxygen polyhedra with temperature or composition projects into the variation of melt density or viscosity, and consequently manipulates the nucleation process^33–35^. (2) Heredity of structural features from melt to crystalline phases. It is demonstrated that the topologically order structures (cations–oxygen polyhedra or the chains/rings connected by these polyhedra) act as prototypes of crystal nucleus^36–39^. In view of these preceding work, it is convincing that elaborative analyses on the structural features of cation–oxygen polyhedra and their variation with temperature and composition will contribute to unveiling the mechanism of polymorphic selection during the crystallization process of titanates from atomic/molecular scale.

BT is a very important functional material from the titanate family. It has several polymorphs and undergoes a sequence of phase transitions during cooling: hexagonal (>~1698 K) → cubic (1698−403 K) → tetragonal (403−273 K) → orthorhombic and rhombohedral (<273 K)^40^. Polymorphic selection of BT has been found during the crystallization process from its undercooled melt and amorphous film, which plays a critical role in preparation of ferroelectric materials, polarized quasi-amorphous films, and colossal permittivity materials^2,41,42^. Here we focus on identifying when and how polymorphic selection takes place during the crystallization process of undercooled BT melt using aerodynamic levitation (ADL) facilities combined with in situ time-resolved HEXRD. We track the structure evolution of BT melt from above the liquidus to supercooled state and reveal an anomalous structure transition in molten BT during freezing across the cubic–hexagonal transition temperature (*T*_c→h_), which accounts for the multi-path crystallization behavior of BT and enables nucleation selection by manipulating the undercooling. The coupling relation between structural transition in melt and nucleation path selection revealed in this work provides a new sight on the elusive crystallization behavior of titanates beyond the traditional cognition of structural heredity, and can be used as a potential strategy to prepare functional oxides with desired phase to meet the property requirement from different applications.

## Results

### Polymorphic nucleation selection manipulated by undercooling

**Fig. 1:**
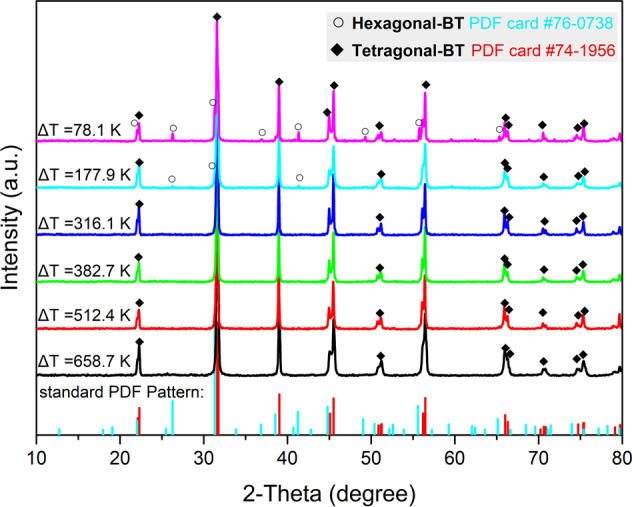
Polymorphic selection behavior of solidified BT. Room-temperature XRD patterns of the solidified BT obtained by triggering nucleation at different undercoolings.

Undercooled BT melt was triggered nucleation at different undercoolings based on ADL facilities. Room-temperature XRD patterns of solidified BT suggest that polymorphic selection occurs during the crystallization process. As shown in Fig. 1, BT crystallizes to pure tetragonal (*t*) phase (*t*-BT, room temperature configuration of cubic phase, *c*-BT) if triggered nucleation at large undercoolings (Δ*T* ≥ 316.1 K), but to a mixture of hexagonal (*h*-BT) and tetragonal phases at small undercoolings (Δ*T* ≤ 177.9 K). The peaks for *h*-BT decrease intensity with increasing undercooling and disappear at the undercooling interval between 177.9 and 316.1 K, which is across the transition undercooling $\Delta T_{{\mathrm{c}} \to {\mathrm{h}}}$ (defined as $\Delta T_{{\mathrm{c}} \to {\mathrm{h}}} = T_{\mathrm{m}} - T_{{\mathrm{c}} \to {\mathrm{h}}}$, where $T_{\mathrm{m}}$ is the melting point and $T_{{\mathrm{c}} \to {\mathrm{h}}}$ is the cubic–hexagonal phase transition temperature, 193 K).

Cross-sectional scanning electron microscopy (SEM) images combined with Raman spectroscopy mapping reveal that *h*-BT and *c*-BT show a regional distribution in the sample with hybrid phases composition (triggered nucleation at Δ*T* = 71 K), as presented in Supplementary Note 1 and Figs. S1 and S2. *h*-BT forms near the top, and *c*-BT generates mainly around the chill contact region, suggesting that nucleation of *h*-BT and *c*-BT requires notably different thermal conditions. Detailed descriptions of the microstructural features at three selected areas (top, middle, and bottom) of the sample are given in Supplementary Note 1. Combining microstructural analysis and XRD patterns, it is plausibly elucidated that the solidified phases selection in BT crystallization is regulated by a polymorphic nucleation sequence manipulated by undercoolings based on the following considerations: (i) *h*-BT and *c*-BT nucleate from the undercooled melt directly rather than from a solid-state phase transition due to the clear trajectory of crystal growth from melt as presented in Fig. S1; (ii) formation of *h*-BT is not cross-nucleation based on *c*-BT crystal nucleus, although this heterogeneous nucleation between cubic and hexagonal phases is common in several condensed matter systems^15–17^. Under the heterogeneous nucleation condition, the amount of *h*-BT should increase with increasing undercooling, which conflicts with the XRD results that the peak intensity of *h*-BT decreases with increasing undercooling (Fig. 1); (iii) precipitation of *c*-BT as prominent phase in supercooled liquid is not due to a higher crystal growth rate, as the crystal growth of both *h*-BT and *c*-BT presents typical facet growth mode (Fig. S3); in addition, the dynamic factors usually affect the selection of phases with different chemical compositions^43,44^.

**Fig. 2:**
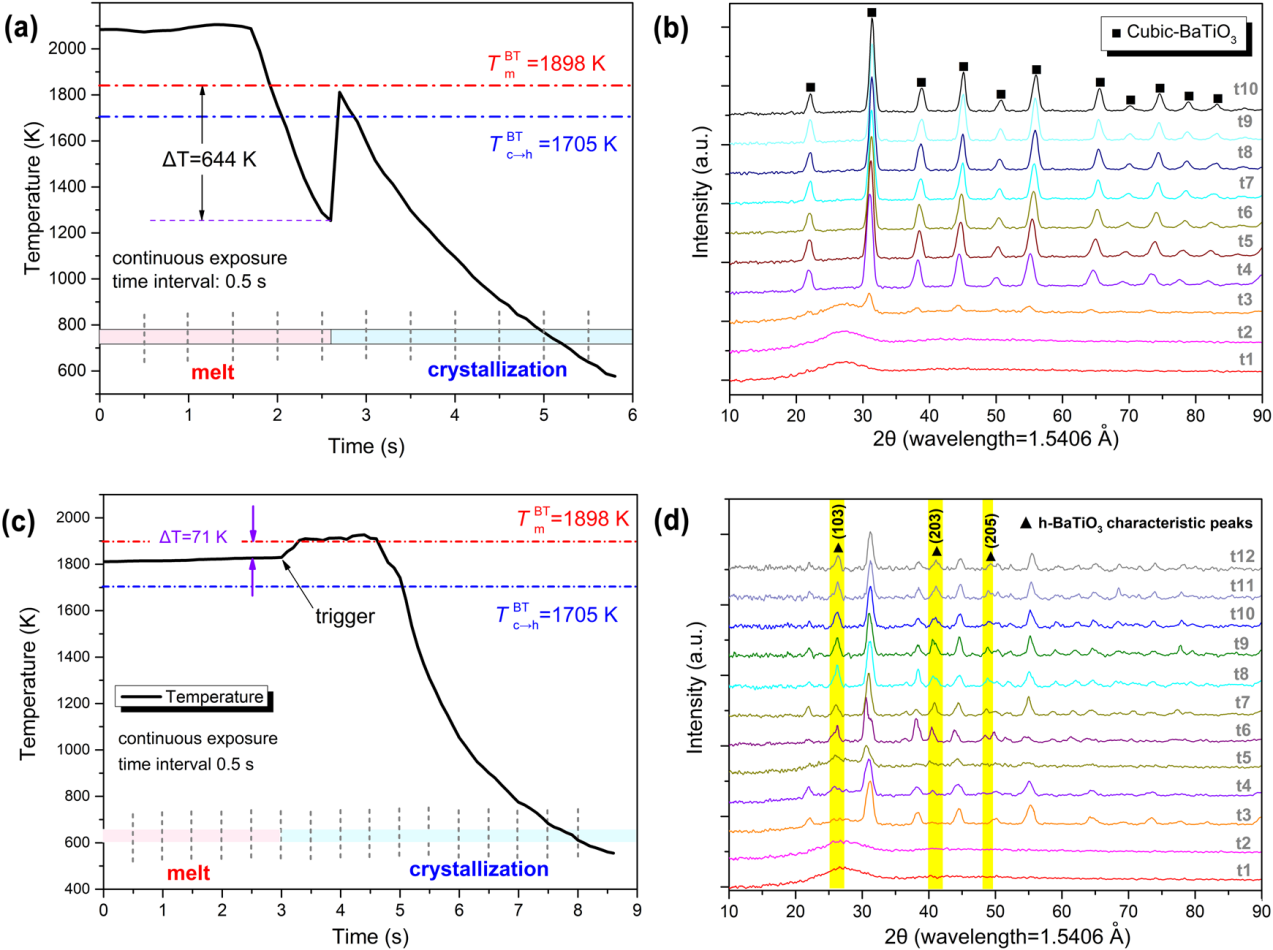
Temperature–time profile and in situ HEXRD patterns describing the crystallization process of BT melt. **a**, **b** Crystallization in supercooled condition (Δ*T* = 644 K). **c**, **d** Being triggered at a small undercooling (Δ*T* = 71 K). The highlight area represents the characteristic diffraction peaks of *h*-BT at 26.28°, 41.22°, and 49.10°. The raw data of 2*θ*-intensity obtained by high-energy X-ray (*λ* = 0.17835 Å) was converted into standard patterns, corresponding to Cu–Kα target (*λ* = 1.5406 Å), for a better comparison with standard PDF cards.

Furthermore, we track the structural evolution of the BT melt during its crystallization process by in situ time-resolved HEXRD. X-ray photon beam is incident on the top-side of the sample and high-angle diffraction signals are collected, as presented in Supplementary Note 2 and Figs. S4–S6. For the supercooled liquid (Δ*T* = 644 K), its temperature–time profile (Fig. 2a) shows a significant recalescence associated with crystallization^45^. Diffraction patterns during the crystallization process of the supercooled liquid obtained from integrating the two-dimensional diffraction patterns (Fig. S5) are presented in Fig. 2b. It is clear that the primary phase solidified from the supercooled liquid is *c*-BT (*t*3), which grows up rapidly without transition to *h*-BT as confirmed by the unvaried diffraction patterns from *t*4 to *t*10. For the crystallization at a small undercooling (Δ*T* = 71 K), the temperature–time profile and the integrated diffraction patterns are shown in Fig. 2c and d, respectively (see Fig. S6 for two-dimensional diffraction patterns). It is intriguing to notice that the primary phase shows up at *t*3 is *c*-BT, suggesting that *c*-BT crystallizes first near the triggering point, which is consistent with the microstructural analysis (Fig. S1 and S2). Subsequently, the characteristic peaks of *h*-BT at 26.28° and 41.22° emerge (*t*4), indicating that *h*-BT has nucleated from small undercooling melt at the upper side of the droplet. The result also provides evidence that *h*-BT is formed from the melt directly rather than from a solid-phase transition from *c*-BT due to the sluggish kinetics for *c* to *h* phase transition which usually takes hours^46^. It is noticed that the diffraction peaks are slightly weakened at *t*5, suggesting the occurrence of re-melting due to release of latent heat. Subsequently (*t*6 to *t*12), the characteristic peaks of *h*-BT are intensified and a fine peak at ~49.10° appears, indicating further growth of *h*-BT. In situ XRD patterns illustrate how BT melt with a small undercooling crystallizes to a mixture of *h* and *c* phases.

Combining the information from microstructural observations and in situ time-resolved HEXRD results, it can be concluded that the polymorphic crystallization of BT stems from the nucleation selection manipulated by undercooling.

### Structural evolution of BT melt

**Fig. 3:**
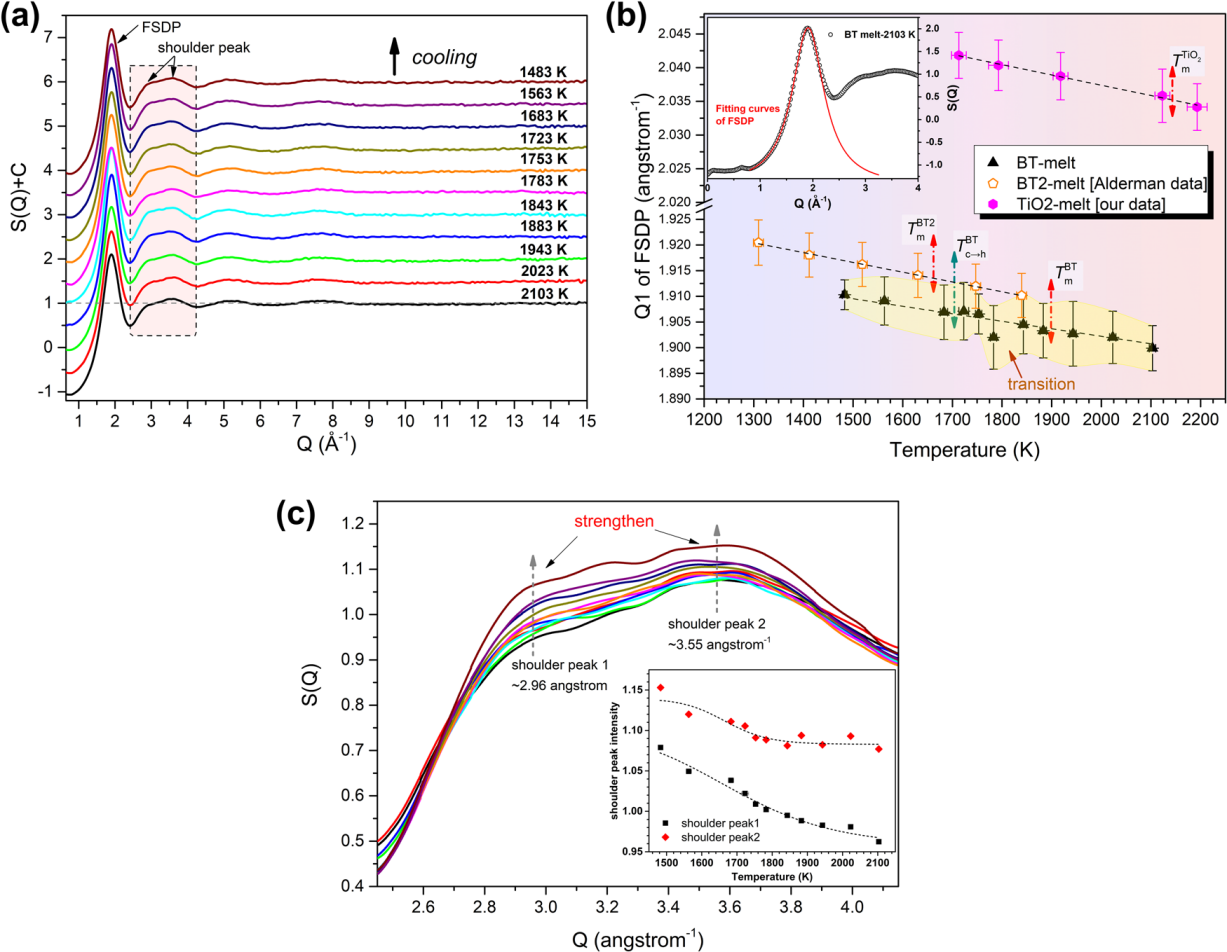
Reciprocal-space structural evolution of the BT melt during cooling. **a** Faber–Ziman structure factors, *S*(*Q*), of molten BT at different temperatures. Note: FSDP represents the first sharp diffraction peak. **b** Variation of the FSDP position as a function of temperature of the BT melt. Data for molten TiO_2_ and BaTi_2_O_5_ melt^47^ are included for comparison. The inset figure is the Lorentzian fitting for FSDP of *S*(*Q*) at 2103 K. Peak position and its associated error bar of FSDP were extracted by the following strategy: the peak position at its maximum intensity is read directly and denoted as *Q*_1_ʹ, and that obtained by Lorentzian fitting is denoted as *Q*_1_ʺ. *Q*_1_ and its uncertainty are defined as |*Q*_1_ʹ+*Q*_1_ʺ|/2 and |*Q*_1_ʹ−*Q*_1_ʺ|/2, respectively. **c** An enlarged view of the second peak in the *S*(*Q*) patterns marked by the dashed rectangle. The inset figure shows the variation of the intensity of two shoulder peaks with temperature.

To explore the structural origin of this nucleation path selection manipulated by undercooling, the melt-BT structure from superheating to supercooling state was tracked by HEXRD. The Faber–Ziman structure factor, *S*(*Q*), of melt-BT at various temperatures is presented in Fig. 3a. *S*(*Q*) of molten BT shows a principal sharp peak at ~1.9 Å^−1^, which reflects the structural information of cation–cation periodicity in the real space (~3.3 Å). The first peak position, *Q*_1_, is commonly used to probe the structural evolution or dynamic transition^47–49^. Here we extracted the *Q*_1_ value and its associated uncertainty by direct reading or Lorentzian curve fitting (inset figure in Fig. 3b). It is intriguing to note that *Q*_1_ shows a discontinuous variation with decreasing temperature that an unanticipated drop is observed (see Fig. 3b) between the phase transition temperature (*T*_c→h_ = 1705 K) and the melting point (*T*_m_ = 1898 K). Such anomalous transition in supercooled BT melt is rarely observed in analogous titanate systems, such as BaTi_2_O_5_ (BT2)^39,47^ and TiO_2_ melt (our data), where the *Q*_1_ increases monotonically with decreasing temperature, as also shown in Fig. 3b for comparison.

This *Q*_1_ anomalous transition is strongly correlated with the variation of rheological properties, which may encode the freezing fate of BT melt. According to the Ehrenfest relation *Q*_1_ = 1.23(2 × *π*/*a*), *Q*_1_ is proportional to the reciprocal of the mean atomic spacing ‘*a*’, which is directly related to the free volume in liquid. Based on Doolittle’s theory^50^, the drop of *Q*_1_ suggests a decrease in the viscosity (*η*) of the melt-BT. Nucleation in cooling BT melt will be therefore greatly promoted due to an enhanced nucleation driving force and a low viscosity, which may be the reason why BT is difficult to vitrify^41^.

The first principal peak embodies intermediate range order (IRO) cation–cation periodicity, which is constructed by short range order (SRO) cation–oxygen polyhedra. The anomalous structural transition of *Q*_1_ prompts us to investigate the structural evolution features of the second diffraction peak that reflects SRO polyhedra in real space (estimated by periodicity 2*π*/*Q*_2_). The second diffraction peak, as shown by the expanded view in Fig. 3c, consists of two shoulder peaks, the approximate positions of which are indicated by the short-dash arrows. We extracted and plotted the intensity of the two shoulder peaks as a function of temperature, as shown in the inset figure in Fig. 3c. It is demonstrated that the intensity of both peaks increases with decreasing temperature but the enhancement of two shoulder peaks are not consistent. There is a relatively larger intensity difference between two shoulders in high-temperature BT melt than that in deep-undercooled melt. Near the structural transition temperature (1783 K) of *Q*_1_, the intensity discrepancy between two shoulder peaks has taken a turning, which strongly indicates that the structure of fundamental motifs (cation–oxygen polyhedra) in BT melt have also changed in cooling process.

**Fig. 4:**
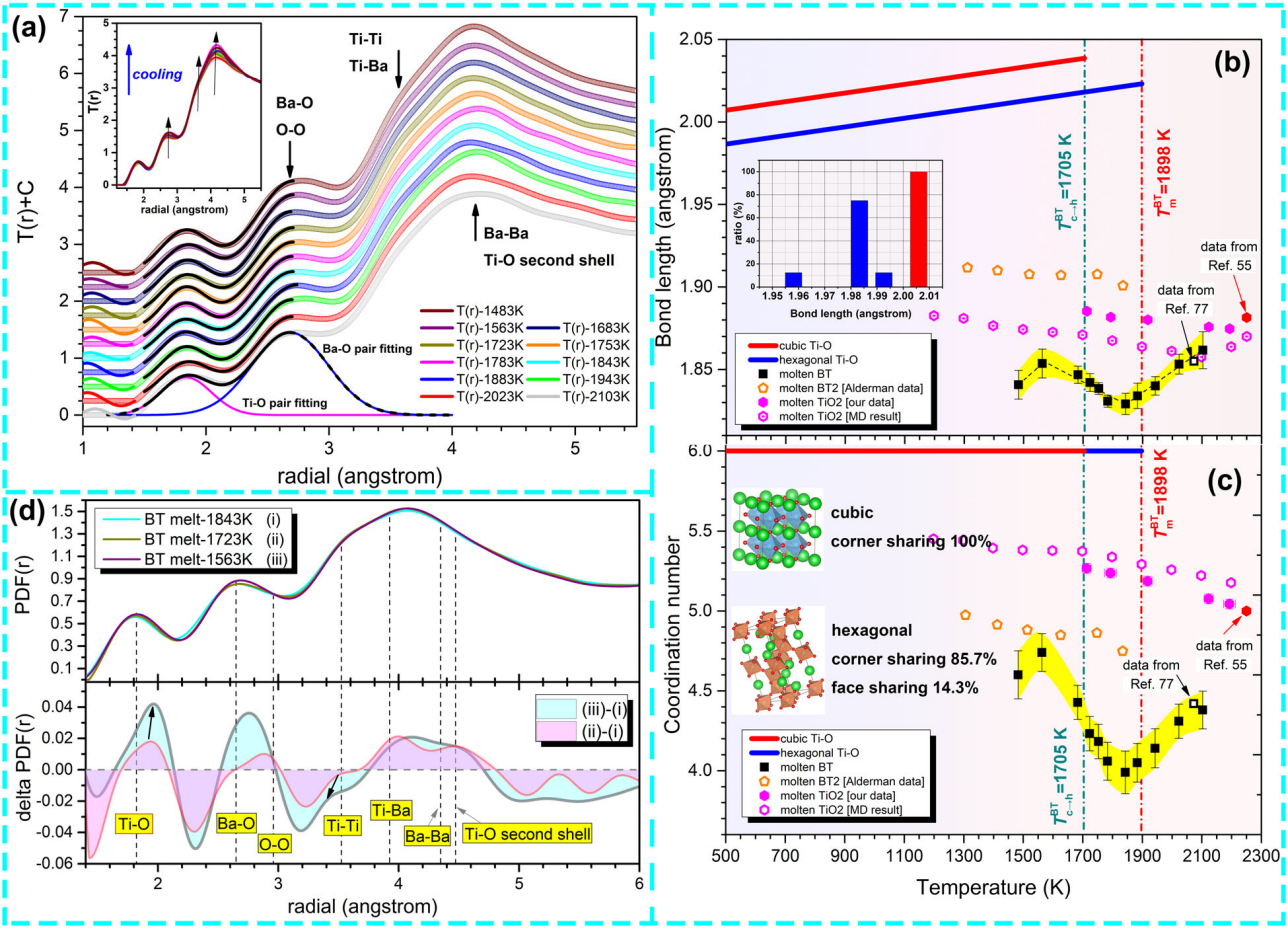
Anomalous structural transition of Ti–O polyhedra in BT melt. **a** The total distribution function $T\left( r \right) = 4{\pi}\rho _0rg(r)$ of the BT melt at different temperatures, *g*(*r*) is pair correlation function. The local interatomic structures of Ti–O and Ba–O were extracted by peak differentiation. The position of other atomic pairs labeled in this figure refers to a previously published work^77^. The inset figure shows the *T*(*r*) patterns without offset along *y* axis. **b** Variation of the Ti–O pair bond length in titanates (BT, BT2 (ref. ^47^), and TiO_2_ (ref. ^55^)) melt with temperature. The error bar corresponds to the mean absolute error of that calculated from the real Ti–O bond length (read directly from *T*(*r*) pattern) and that obtained from Gaussian fitting. The Ti–O bond length of *c*-BT and *h*-BT calculated by using weighted average are also presented for comparison. The inset graph gives the Ti–O bond length distribution in *c*-BT and *h*-BT unit cell, respectively. **c** The coordination number of Ti–O pair in BT and other titanate melts (BT2 (ref. ^47^), TiO_2_ (ref. ^55^)) at different temperatures. The error bar represents the mean absolute error of two Ti–O coordination number results estimated by integrating the original *T*(*r*) curves from 1.4 Å to the first minima and by integrating the Gaussian fitting curves. Ti–O octahedra motifs (CN = 6) in crystalline phases are also included for demonstration. Open squares represent the measurement by Alderman et al.^77^ for BT superheated melt. **d** The change of pair distribution functions across the structural transition process (upper), and the corresponding difference functions (lower). The vertical dash lines are used to indicate the approximate bond length of each atomic pair. These values are referred from ref. ^77^ and our AIMD results.

The anomalous behavior observed in reciprocal space is mirrored in the structural evolution of SRO cation–oxygen polyhedra, which can be illustrated by the total distribution function *T*(*r*) of BT melt. Bulk density of the suspended BT droplet required for calculating *T*(*r*) is re-measured by combining ADL with an ultraviolet-based imaging technique (see details in Supplementary Note 3 and Figs. S7, S8). The structural parameters of two types of cation–oxygen polyhedra in the BT melt, Ti–O and Ba–O polyhedra, can be extracted by differentiating and fitting the first two peaks on the *T*(*r*) curves, as shown in Fig. 4a. Considering the large difference in ionic radii of Ti^4+^ (0.53 Å, five-fold) and Ba^2+^ (1.36 Å, six-fold)^51^, the first peak on the *T*(*r*) pattern (<2 Å) is mainly attributed to the Ti–O pair; the second peak ranging from 2.10 to 3.05 Å reflects overlapping information from O–O and Ba–O pairs assuming the ionic radius of O^2−^ is 1.35 Å. Variation of the Ti–O bond length in molten BT with temperature is shown in Fig. 4b. It can be seen that the Ti–O bond length first decreases with decreasing temperature, but this trend reverses in the temperature range across *T*_c→h_ that it increases from 1.83 Å (1843 K) to 1.85 Å (1563 K). Such discontinuous variation of bond length is rarely observed in molten oxides^52–54^ including titanates such as molten TiO_2_ (ref. ^55^) and BT2 (refs. ^39,47^), in which the bond length of cation–oxygen pairs varies monotonically with decreasing temperature. Furthermore, when liquid BT is undercooled into cubic region (*T* < *T*_c→h_), the Ti–O bond length is significantly larger than that extrapolated from the high-temperature data. Based on the bond valence theory^56^, a large Ti–O bond length indicates that Ti cations are bonded with more anions (O^2−^), and thus an enhanced connectivity of Ti–O polyhedra in the BT melt.

The coordination number, $C_{{\mathrm{Ti}}}^{\mathrm{n}}({\mathrm{O}})$, of the Ti–O pair is calculated from *T*_n_(*r*) using the same method reported by Hennet^57^.

1 $${\int}_{r_1}^{r_2} {rT_{\mathrm{n}}(r)dr = \frac{{W_{{\mathrm{Ti}},{\mathrm{O}}}}}{{c_{\mathrm{o}}}}C_{{\mathrm{Ti}}}^{\mathrm{n}}({\mathrm{O}})}$$

where *r*_1_ and *r*_2_ are the minimum and maximum cutoff of the Ti–O pair, which are set as 1.50 and 2.75 Å, respectvely; *c*_o_ is the concentration of oxygen atoms and $W_{{\mathrm{Ti}},{\mathrm{O}}}$ is the weighting factor of Ti–O pairs (see details in Supplementary Note 4 and Fig. S9). The calculated $C_{{\mathrm{Ti}}}^{\mathrm{n}}({\mathrm{O}})$ is plotted as a function of temperature, as shown in Fig. 4c. It is observed that the Ti–O coordination number initially decreases with decreasing temperature, but this temperature dependence reverses in the temperature range between 1843 and 1563 K (across *T*_c→h_). Such a transition is extraordinary in comparison with the continuous variation of coordination number in TiO_2_ melt^55^ and BT2 melt^39,47^. This discontinuous structural evolution of Ti–O motifs is rare and poorly understood in previous structural studies of molten titanates. In order to further unveil the detailed scenario of the structural changes, the pair distribution functions (PDFs) within the temperature range of structural transition and the corresponding difference functions are extracted and compared in Fig. 4d. It is found that the Ti–O peak shifts to higher *r* during transition, being concomitant with an increased intensity and FWHM, which indicates an increase in the percentage of Ti–O polyhedra with high oxygen coordination number. This conclusion is also consistent with the $C_{{\mathrm{Ti}}}^{\mathrm{n}}({\mathrm{O}})$. Estimating from the electrostatic bond strength theory, the increased oxygen coordination number surrounded Ti cations implicates that more Ti–O polyhedra in the melt are interconnected by means of oxygen sharing. Consequently, the enhanced connection between Ti–O polyhedra may contribute to the strengthened correlation of pertinent atomic pairs, such as O–O and Ti–Ti, which are manifested by enhancement of relevant peaks *T*(*r*) patterns (inset figure in Fig. 4a). Additionally, it is intriguing to note that the correlation length of Ti–Ti shrinks with the structural transition proceeding, as shown by the difference functions Fig. 4d, which suggests that the connection mode between some Ti–O polyhedra has perhaps changed from long connection (corner sharing) to short connection (edge sharing or even face sharing).

**Fig. 5:**
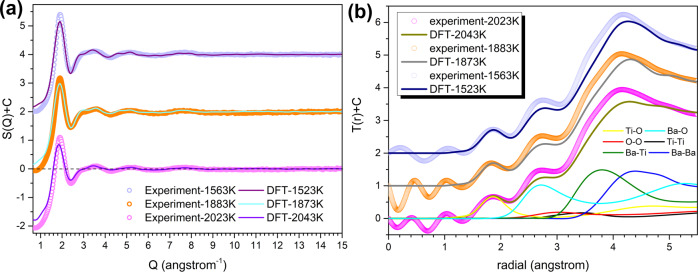
The comparison of structural correlation functions determined by AIMD simulation and experimental measurement. **a** Faber–Ziman structural factors, *S*(*Q*); **b** total distribution functions, *T*(*r*). In order to manifest the contribution of each partial atomic pairs on total scattering, the partial *T*_ij_(*r*) functions corrected by real-space X-ray weighting factors, $W_{{\mathrm{ij}}}(r) \otimes T_{{\mathrm{ij}}}(r)$, are also presented in **b**. Note that the $W_{{\mathrm{ij}}}(r)$ is the Fourier transform of $W_{{\mathrm{ij}}}(Q)$, and $\otimes$ represents convolution operation.

**Fig. 6:**
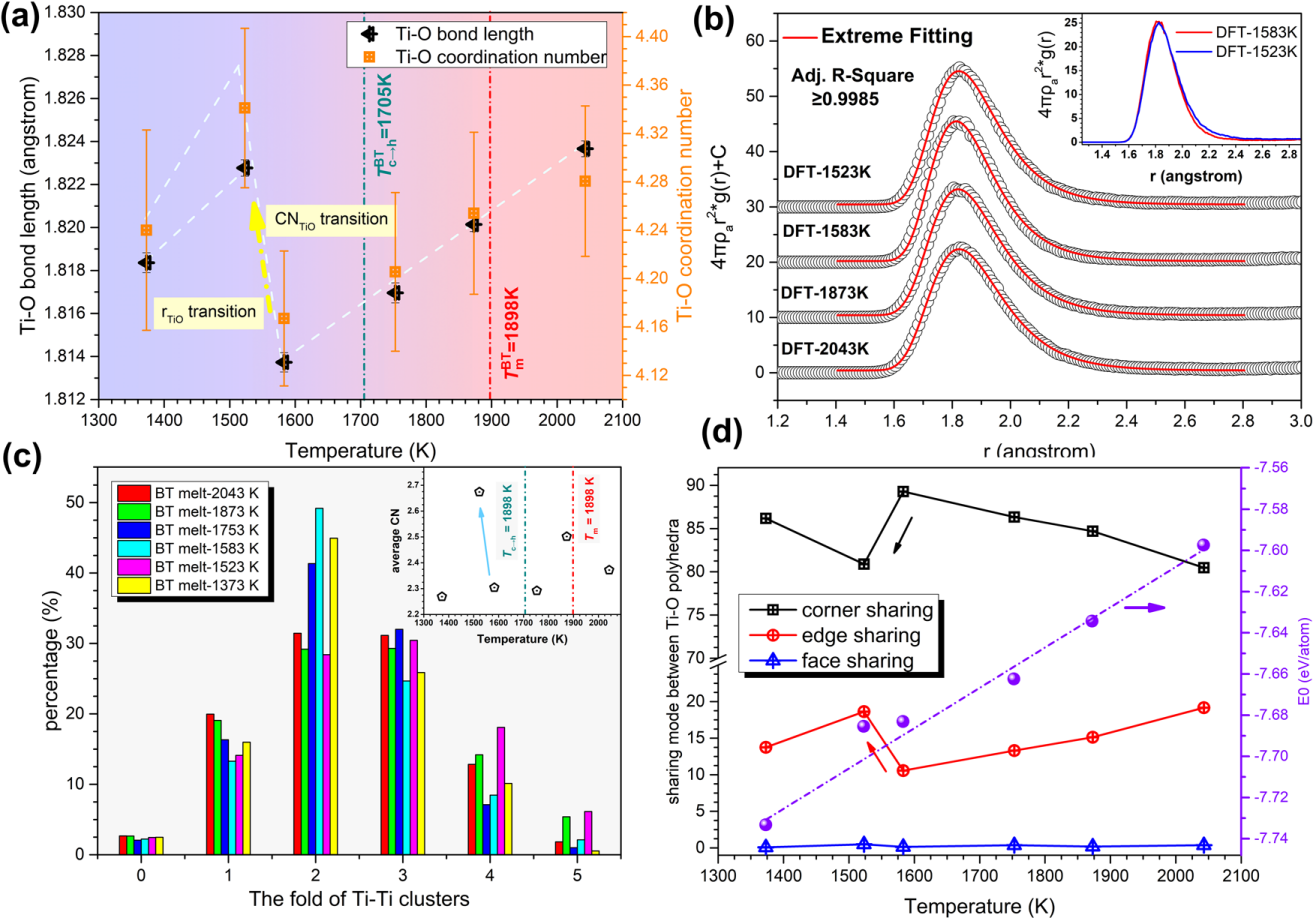
The structural transition scenario embodied by AIMD ensembles. **a** Temperature dependence of Ti–O polyhedra structural parameters. The error bars correspond to the standard deviation of Ti–O coordination number based on 5000 configurations. **b** Comparison of the Ti–O partial radial distribution function (RDF) before and after the structure transition. **c** Variation of the fold of Ti–Ti clusters. The inset figure presents the variation of Ti–Ti total coordination number with temperature. **d** Connectivity between Ti–O polyhedra as a function of temperature; in addition, the variation of average energy per atom (*E*_0_) with temperature is also plotted in this figure.

In view of the Ti–O polyhedra transition behavior in Fig. 4b and c, it is elucidated that the structural transition occurring in undercooled BT melt may be driven by temperature, namely, gradual transformation through incremental metastable states within a certain temperature range. This is analogous with several first-order phase transition behaviors (e.g. high-/low-density amorphous transition in glassy water^35^). Given the controversial and complicated nature of liquid–liquid structural (phase) transition, we perform ab initio molecular dynamics (AIMD) simulations to give further evidence to support the experimental observations. The comparison of structural correlation functions determined by AIMD simulation and measured by experiment, at three typical temperatures (superheating, near melting point, and supercooling melt), is shown in Fig. 5. It is intuitive that the total *S*(*Q*) functions obtained by AIMD reflect all the characteristic peaks in corresponding with experimental patterns (see Fig. 5a); however, strong finite size effect of AIMD boxes may lead to the deviation of *S*(*Q*) patterns in low-*Q* side and the first principal peak. The comparison of *T*(*r*) functions in real space is presented in Fig. 5b. The first Ti–O peak shows a good accordance between simulation and experiment, but the second peak and the third peak in simulation patterns are relatively lower than that of experimental patterns, which may correspond to a lower FSDP in simulated *S*(*Q*) patterns, because this peak is usually regarded to be related to IRO cation–cation periodicity (the third peak in *T*(*r*) patterns). The contribution from different atomic pairs on total *T*(*r*) functions was also presented by Fig. 5b, it is clear that the first peak in *T*(*r*) pattern is dominant by Ti–O pair, and the second peak is mainly contributed by Ba–O pair. The overlap between Ti–O peak and Ba–O peak is not serious, benefiting from a large difference of ionic radius between Ti^4+^ and Ba^2+^, which indicates the peak-splitting and fitting in Fig. 4b and c is reasonable. Structural statistics given by AIMD are presented in Fig. 6. In the rapid quenching of BT melt, it is illustrated that both the bond length and the coordination number of Ti–O pair show a discontinuous transition in a narrow temperature range (1523–1583 K), as presented in Fig. 6a. The temperature dependence of Ti–O polyhedra structural parameters calculated by AIMD is consistent with our diffraction experimental results (Fig. 4b and c) apart from a difference in the transition temperature. One possible reason we considered is that this anomalous structural transition is strongly dynamic-correlated, and it will be influenced by thermal history just like most phase transitions in materials. The typical partial radial distribution functions (RDFs) of Ti–O pair are presented in Fig. 6b, the intensity of Ti–O RDF patterns in high-*r* side increases after structural transition (inset graph in Fig. 6b), which corresponds to a larger coordination number and is consistent with our experimental results (Fig. 4d). Intriguingly, the Ti–O RDF patterns shown asymmetric feature, which can be fitted by asymmetric Extreme function appropriately, but is somewhat against to before Gaussian peak fitting (see Fig. 4b). The speculation that enhanced connection between Ti–O polyhedra and the change of connection modes are also confirmed, as presented in Fig. 6c and d. After structural transition, the folds of Ti–Ti clusters are notably increased, indicating the polymerization between Ti–O polyhedra is strengthened. Additionally, after transition, the percentage of corner sharing mode decreases, in contrast, the edge sharing mode increases, which accounts for the contracted Ti–Ti correlation length in Fig. 4d. The change of connection between Ti–O polyhedra also corresponds to an essential fluctuation of average energy per atom (*E*_0_), as shown in Fig. 6d. Due to the percentage of unstable edge sharing increases after structural transition, a small positive deviation of *E*_0_ is observed, which also provide firm evidence of structural transition in BT supercooled liquid from perspective of energy.

**Fig. 7:**
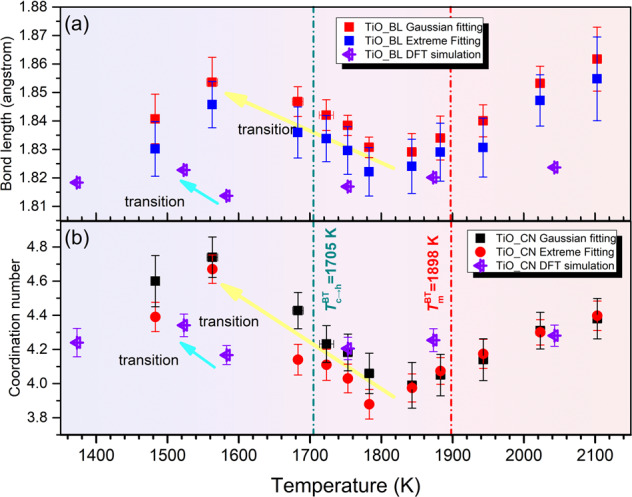
Comparison of the anomalously structural transition revealed by diffraction experiment and AIMD simulation. **a** The bond length of Ti–O pairs and **b** the coordination number of Ti–O polyhedra. Two peak fitting methods was used to deal with the first Ti–O peaks in experimental *T*(*r*) patterns, and the extracted structural parameters are compared in this figure. Definition of error bars is the same as described in captions of Figs. 4b, c and 6a.

Given the asymmetric feature of Ti–O RDF peaks illustrated by AIMD, we compared the difference of Ti–O structural parameters obtained by Gaussian or Extreme curves fitting, as shown in Fig. 7. In addition, the size of structural transition determined by AIMD simulation and diffraction experiment were also compared in this figure. The bond length and coordination number of Ti–O pair obtained by different peak fitting method are quite accordant with each other, which manifest the structural transition observed in Fig. 4b and c should not be so-called uncertainties introduced by Gaussian peak fitting. Although the structural transition determined by AIMD simulation is smaller than that observed in experiment, the transition size is in the same order magnitude. In addition, the size of structural transition determined by AIMD is clearly exceeding the standard error caused by configurational fluctuation, therefore, a convinced structural transition in supercooled BT liquid is expected from AIMD evidence. Furthermore, we also test the discontinuous transition of Ti–O coordination number determined by AIMD, using a different starting configuration or even a completely random starting configuration (see details in Supplementary Note 5 and Fig. S10). The anomalous structural transition existing in undercooled BT liquid are seemingly independent of starting configuration. However, it should be emphasized that the magnitude of structural transition determined by AIMD may be sensitive to the total energy state of starting configurations, a relatively stable starting configurations with lower total energy may be helpful to investigate this liquid–liquid structural transition.

## Discussion

### Relationship between polymorphic nucleation selection and structure heredity

The structural tracking reveals an anomalous structure transition of undercooled BT melt during the freezing process, which may promote nucleation. In the following sections, we would like to discuss the question whether the polymorphism of BT melt accounts for the subsequent polymorphic crystallization selection. Both the classical nucleation theory (CNT) and the more widely accepted multistep nucleation scenario suggest that a crystal with structure analogous with the parent (liquid) phase possess a low nucleation barrier^58,59^, which is known as structural heredity. In previous studies, evolution of the SRO cation–oxygen polyhedra in molten oxides is regarded as an essential structural probe to describe liquid–solid transition^38,60^, glass forming^32^, and liquid–liquid phase transition^48,49^ because connection of these SRO structural units constructs larger order structure, such as IRO or critical nucleus. Therefore, we first take the SRO cation–oxygen polyhedra into consideration.

In titanates, Ti–O polyhedra are regarded as the structural units because Ti–O pair has stronger bonding strength than Ba–O. Thus, the structural parameters of Ti–O polyhedra in melt-BT with those in crystalline *c*- and *h*-BT phases are compared. The mean Ti–O bond length in crystalline phases is calculated based on published crystal configurations (*c*-BT^61^, *h*-BT^62^) by extrapolating the available data to high temperature range assuming the same thermal expansition coefficient. Coordination number of the Ti–O pair in *c*- and *h*-BT is 6 and remains unvaried with temperature. As shown in Fig. 4b and c, both bond length and coordination number of Ti–O pair in the BT melt are significantly lower than those in the crystalline phases. The striking difference in the structral parameters between BT melt and crystal phases demonstrates that the crystal structure is severely destroyed during the melting process, and suggests that BT liquid is very fragile^32^. It is also noticed that the Ti–O bond length in BT melt is closer to that in *h*-BT than in *c*-BT through the freezing process, indicating that the Ti–O polyhedra in BT melt have a higher degree of similarity with those in *h*-BT than in *c*-BT, which cannot explain the preferential nucleation of *c*-BT in supercooled liquid.

**Fig. 8:**
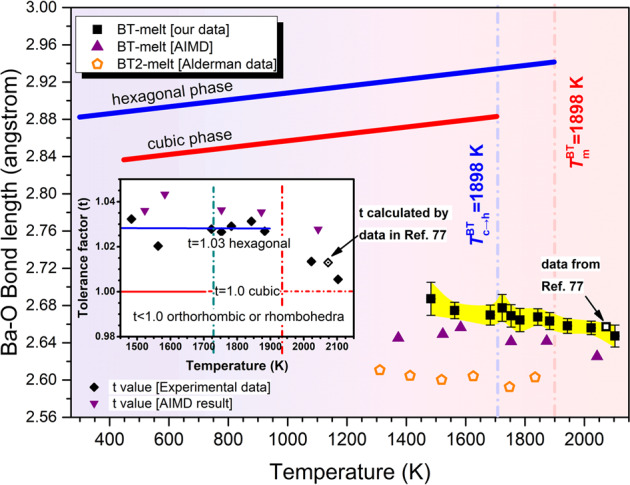
Structural evolution of Ba–O polyhedra in BT melt during cooling. The variation of Ba–O mean bond length in BT melt, obtained from peak-differentiation of *T*(*r*) or calculated based on AIMD ensembles, as a function of temperature. The error bars correspond to the standard deviation derived from Gaussian fitting. Open square is the result from ref. ^77^. Data for BT2 melt^47^ and two crystalline BT phases^61,62^ are also included for comparison. The inset figure shows the tolerance factor $\left( {t = \frac{{d_{{\mathrm{Ba}} - {\mathrm{O}}}}}{{\sqrt 2 d_{{\mathrm{Ti}} - {\mathrm{O}}}}}} \right)$ of BT melt and crystalline phases at different temperatures.

On the other hand, the bond length of Ba–O pair is also extracted by peak-differentiating and plotted as a function of temperature, as illustrated in Fig. 8. For comparison, the calculated Ba–O bond lengths from AIMD simulations are also presented. Different from Ti–O pair, Ba–O pair does not show abrupt variation across *T*_c→h_. In addition, the Ba–O bond length in BT melt is closer to *c*-BT rather than *h*-BT. These conflicting information from Ti–O and Ba–O suggests that SRO of cation–oxygen polyhedra is inadequate to describe the polymorphic nucleation selection of BT.

On a larger scale, we investigate the tolerance factor of the undercooled BT melt. The tolerance factor, defined as $t = d_{{\mathrm{A}} - {\mathrm{O}}}/\sqrt 2 d_{{\mathrm{B}} - {\mathrm{O}}}$ where *d* represents the bond length of cation–oxygen^63^, describes the structure stability of ABO_3_-type perovskites; *t* has been successfully used to explain the stabilization of metastable phases and nucleation behavior in RFeO_3_ (ref. ^64^) and RMnO_3_ (ref. ^65^) (R = rare-earth element) prepared by containerless processing under low oxygen partial pressure. For BT, *t* = 1 suggests a cubic structure and *t* = 1.03 suggests a tetragonal or hexagonal phase. The calculated *t* of the undercooled BT melt shows a fluctuation around 1.03 (inset figure in Fig. 8), which agrees with the AIMD simulation results and suggests that the melt structure has a high similarity with the high-temperature *h*-BT phase. From the traditional view of structural similarity, BT melt should crystallize to *h*-BT, which fails to explain the polymorphic nucleation of undercooled BT melt. This drives us to explore more basic generality on the polymorphic nucleation selection of BT.

### New insight of BT polymorphic nucleation selection based on polyhedra polymerization

**Fig. 9:**
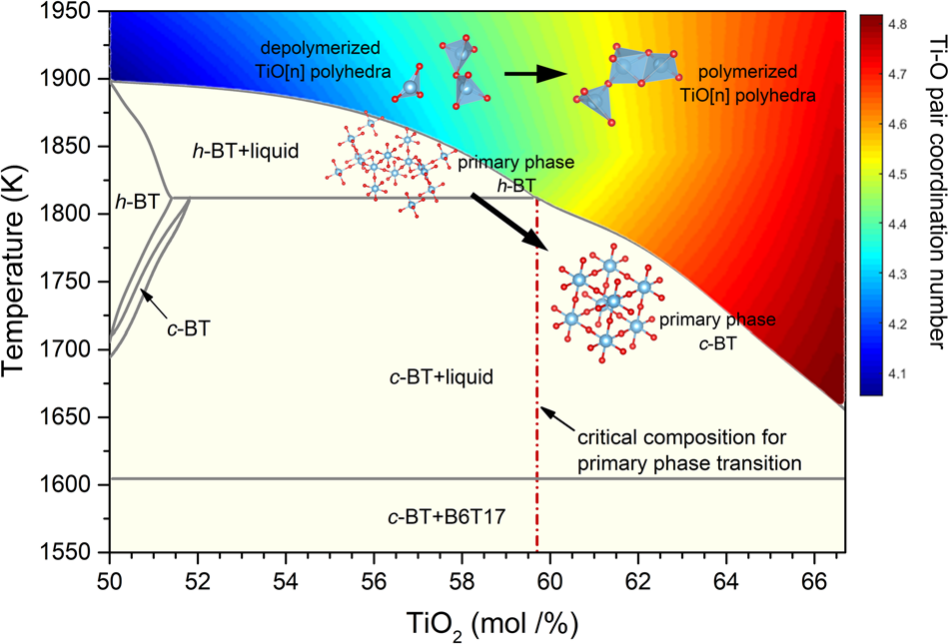
Parallel effect of anomalous structure transition of molten BT near *T*_c→h_ with increasing TiO_2_ content in equilibrium state. Variation of structural polymerization (demonstrated as mean Ti–O coordination number) in (1−*x*)BaO−*x*TiO_2_ (*x* from 50% to 66.67%) melt with both composition and temperature. Phase diagram of BaO–TiO_2_ binary system in the composition range of 0.5 ≤ *x* ≤ 0.6667 is shown below.

Previous studies suggest that bridging between Ti–O polyhedra plays an important role in the choice of crystallization path. For instance, weakening the bridging by oxygen vacancies^66^ is responsible for the retention of metastable *h*-BT in the sintering process, whereas restricting the arrangement of Ti–O polyhedra (strengthening the bridging) directly regulates the polymorphic selection during the crystallization in amorphous BT film^42^. These facts inspire us to explore the possible interrelationship between bridging of Ti–O polyhedra and polymorphic nucleation selection of BT. Bridging of cation–oxygen polyhedra can be described by a polymerization, which is defined as the ratio of non-bridging oxygen per cation and is approximately delineated by the mean oxygen coordination number around cations in here. Polymerization has been employed to understand the dynamic behavior^67^, glass forming ability^36^, and crystallization^52^ of strong molten oxides such as silicates, aluminates, and borates. However, for extremely fragile melt such as zirconates and titanates, polymerization is less attended due to distinct structural units in melt and corresponding crystals^32,34,55^. In the BaO–TiO_2_ system associated with this work, polymerization has exhibited a potential influence on polymorphic nucleation: with strengthening Ti–O polyhedra bridging (excess of TiO_2_), the primary phase of BT changes from *h*-BT to *c*-BT^46^. Here we have a quantitative analysis of the possible effect of polymerization on the primary phases crystallized from melt. The evolution profile of Ti–O coordination number with temperature and composition in (1−*x*)BaO−*x*TiO_2_ (0.5 ≤ *x* ≤ 0.6667) melt is calculated by interpolation:

2 $$N_{(1 - x){\mathrm{Bao}} - x{\mathrm{TiO}}_2}^T = \frac{{\left( {0.6667 - x} \right)}}{{0.1667}}N_{{\mathrm{BT}}}^T + \frac{{\left( {x - 0.5} \right)}}{{0.1667}}N_{{\mathrm{BT}}2}^T$$

and illustrated as the color map in Fig. 9 about the BaO–TiO_2_ binary phase diagram^46^. Along the liquidus, the Ti–O coordination number increases with increasing TiO_2_ content (*x*), indicating that the polymerization among Ti–O polyhedra units is strengthened. At the critical composition point for *h* to *c* transition (*x* = 0.596), the Ti–O coordination number is about 4.50, which is comparable to the value of cubic region ($T < T_{{\mathrm{c}} \to {\mathrm{h}}}$) after structural transition (Fig. 4c), suggesting that the variation of polymerization in undercooled BT melt resulted from anomalous structure transition across *T*_c→h_ is parallel to that induced by increasing TiO_2_ content. Therefore, the polymorphic nucleation behavior may be ‘self-regulated’ by the dynamically polymerized and depolymerized tendency between Ti–O polyhedra. Although the scenario of polymerization change induced by varying TiO_2_ content and by manipulating undercooling are different (linear variation with TiO_2_ content whereas non-linear variation with undercooling), they both result in an enhancement in polymerization. It is therefore concluded that the strongly ordered [TiO_*m*_]_*n*_ groups developed in highly polymerized BT melt (large-undercooling or rich-TiO_2_) may initiate the *c*-BT nucleation, in contrast, weak bonded units in liquid (small-undercooling or poor-TiO_2_) will contribute to the preferential crystallization of *h*-BT.

## Conclusion

By in situ HEXRD, we reveal an anomalous structure transition in molten BT during cooling across the cubic–hexagonal transition temperature (*T*_c→h_), which accounts for the multi-path crystallization behavior of BT. Such abnormal transition is rarely observed in other molten oxides and enables nucleation selection of BT by manipulating the undercooling: *c*-BT is preferred if nucleation is triggered at a large undercooling ($T \,< \,T_{{\mathrm{c}} \to {\mathrm{h}}}$), whereas *h*-BT precipitation is promoted at a small undercooling ($T_{{\mathrm{c}} \to {\mathrm{h}}} \,< \,T \,< \,T_{\mathrm{m}}$). We find that the nucleation selection between *h*-BT and *c*-BT breaks the traditional cognition of structural heredity.

The atomic-scale mechanism behind this polymorphic nucleation selection manipulated by undercooling was investigated by tracking the structural evolution of BT melt from overheating to supercooling state. A discontinuous structure transition mirroring both in reciprocal space correlation function *S*(*Q*) and in the structural parameters of cation–oxygen (Ti–O) polyhedra is observed across *T*_c→h_ during cooling of a BT melt. We unveil that the nucleation selection between *c*- and *h*-BT is self-regulated by the intrinsic structure property of the melt, in particular, the degree of polymerization between Ti–O polyhedra: a highly polymerized liquid promotes the cubic phase nucleation, on contrary, the hexagonal phase primarily precipitates from more depolymerized liquid. Our findings shed light on the atomic-scale scenario of liquid-BT structural evolution, and provide a new perspective (polymerization effect) for linking structural transition occurring in molten oxides with alternation of crystallization path, which will spur further investigation in physical mechanism and industrial application.

## Methods

### Sample preparation and containerless processing

Highly pure BaCO_3_ (99.95%, Aladdin, China) and TiO_2_ (99.99%, rutile, Aladdin, China) powders were used as starting materials to synthesize BT. Equimolar BaCO_3_ and TiO_2_ were homogeneously mixed by wet ball milling in ethanol for 36 h. After drying, the resultant mixture was pressed into tablets (diameter 20 mm, thickness 2 mm) at 20 MPa by uniaxial press, and then sintered in a muffle furnace at 1273 K for 12 h. The sintered tablets were crushed to small pieces of ~15 mg and were used for subsequent containerless processing. The containerless melting was conducted by ADL equipped with a 100 W CO_2_ laser. Samples were suspended in the furnace by purging highly pure oxygen (99.999%) with controlled flow rate to provide drag force and buoyancy to balance the gravity. A CCD camera was used to monitor the suspension status of sample and an infrared pyrometer with a wavelength of 1.255 μm was used to record the temperature profile. Nucleation was triggered by decreasing oxygen flow, which destroyed the stable levitation of the molten droplet and induced the nucleation chilling effect (contact with the wall of nozzle). Once recalescence was observed on the temperature–time profile, laser was turned off immediately to preserve the primary phase by quenching.

### Characterization of phase composition and microstructure

The phases of solidified BT were identified by Cu Kα XRD (Rigaku Ultima IV) on finely ground powder. Spacial distribution of each phase and sample morphology were characterized by Raman imaging combined with SEM (Raman-SEM, MAIA3 GMU model 2016) on the polished and chemically etched cross-section. Raman spectrum was obtained by a 532-nm Ar laser with a spot size of ~0.3 μm.

### In situ time-resolved HEXRD and data processing

**Fig. 10:**
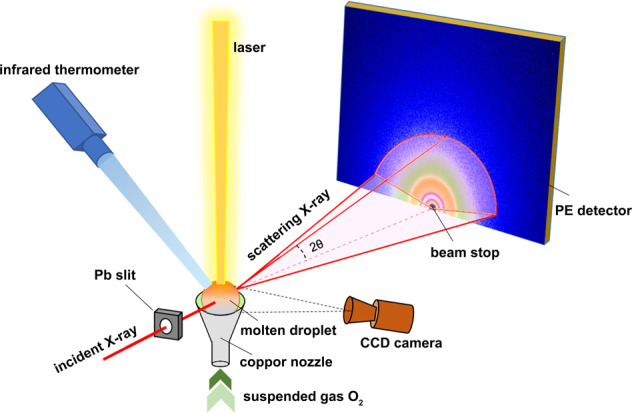
Experimental setup. The schematic diagram of ADL device combined with synchrotron radiation HEXRD.

The diffraction experiments were conducted in beam line station BL13W1 of Shanghai Synchrotron Radiation Facility (SSRF). ADL facilities were assembled on a two-axis translation stage to ensure precise alignment between the specimen and the synchrotron radiation X-ray beam. Schematic of the experimental setup is shown in Fig. 10. The energy of high-energy X-ray was calibrated by a tungsten target as 69.525 KeV (a wavelength of 0.17835 Å), which provides a sufficient scattering range *Q* and reduces the influence of self-absorption and multiple scattering. A PE flat-panel X-ray plate (XRD 1621 AN3 ES) was used to collect the scattering photons during the step cooling process. Data collection time for time-resolved HEXRD experiment (tracking crystallization) was 0.5 s to ensure signal quality.

To characterize the structure of molten BT, data collection time was extended to 60 s (4 × 15 s) to ensure temperature stability and to avoid overexposure of the PE plate. The X-ray beam (1.3 mm × 0.6 mm) was partially incident by the top of the specimen, as shown in Fig. S4, to obtain larger count rates at larger 2*θ* values^68^. Background diffraction was measured on empty chamber without sample.

The initial diffraction intensity, extracted by integrating the image plate pattern through software fit2D, was corrected for polarization, absorption, Compton scattering, and fluorescence. After that, the normalized scattering intensity, *I*s(*Q*) was used to obtain the total X-ray structural factor *S*(*Q*). Subsequently, the distribution functions, such as *T*(*r*) and *g*(*r*), in real space can be derived from *S*(*Q*) through Fourier transition corrected by Lorch function to reduce the truncation effect^69^.

### Density measurement of molten BT droplet

The melt density of BT was measured by combining ADL with ultraviolet-based imaging technique, as described by Langstaff^70^. A schematic diagram of the facility is shown in Fig. S7. A high-resolution, black and white high-speed camera (Vision Research Inc, VEO 640) was used to acquire the magnified image of the sample, which was illuminated by a UV lamp (LC8, L9588-02) from the opposite direction. To eliminate the thermal radiation noise (mainly visible and infrared light) at high temperatures, which may blur the boundary of the droplet, a high-pass filter (Thorlabs, FESH0450) was mounted in front of the high-speed camera lens. The molten droplet is approximately ellipsoidal, and its geometric size was obtained by fitting the boundary of the backlighted image by an elliptic equation. The actual size of a molten droplet was converted by a scale factor, which was determined through fitting the boundary of the backlighted imaging of a SiC bead with known diameter ($\emptyset = 3.175\,{\mathrm{mm}}$).

### AIMD simulations

AIMD calculations were performed by using the density functional theory (DFT) with Vienna AB-initio Simulation Package (VASP)^71^. Interaction between the ionic core and the valence electrons is treated by the projector augmented wave (PAW) method^72,73^. The exchange-correlation interactions were described by the generalized gradient approximation (GGA) in the Perdew Burke Ernzerhof (PBE) formalism^74,75^. A canonical ensemble (constant atomic number, volume, and temperature, NVT) was employed. The cutoff energy was set as 400 eV. The 2 × 2× 2 grid was used for Brillouin zone integrations. An energy tolerance of 10^−5^ eV and a force tolerance of 0.01 eV Å^−1^ were adopted to ensure accuracy. The initial liquid model, containing 200 atoms (40 Ba, 40 Ti, and 120 O), was generated by fitting the experimental structural functions (*S*(*Q*) and *g*(*r*)) based on Reverse Monte Carlo method. This initial starting configuration was relaxed in 5000 K for 30 ps with a timestep of 3 fs, such a high temperature was used to guarantee a non-crystalline configuration. Then, the high-temperature configuration was cooled to the desired liquid temperatures, after that, a simulation time of 15 ps with a timestep of 3 fs was performed to ensure energy stability. At each temperature, the model was equilibrated for another 5 ps with a timestep of 1 fs. The configurations corresponding to the final 5 ps were collected to calculate the partial PDF, the folds of Ti–Ti clusters, the connectivity between Ti–O polyhedra, and other structural parameters. In all the AIMD simulations, temperature was controlled by a Nosé thermostat^76^. The total pressure was obtained from the sum of ideal gas part (ρkBT) and the external pressure was provided by VASP. Under each temperature, the supercell length was tested carefully to ensure that the absolute value of the total pressure is lower than 200 MPa.

## Supplementary information

Supplementary Information

## Data Availability

The Supplementary Information provides detailed description of the microstructure and the regional distribution of *h* and *c*-BT in the samples after solidification (Supplementary Note 1 and Figs. S1–S3). Two-dimensional diffraction patterns recorded in BT crystallization process are shown in Supplementary Note 2 and Figs. S4–S6. The motivation for re-measuring the density of molten BT and the density result as a function of temperature are included in the Supplementary Note 3 and Figs. S7 and S8. Pair weighting factors *W*_ij_ of BT are presented in Supplementary Note 4 and Fig. S9. The robustness of the structural transition determined by AIMD is identified in Supplementary Note 5 and Fig. S10. The data supporting the findings of this study are available from the corresponding author on reasonable request.

## References

[1] Li QJ (2013). Single crystal growth of the pyrochlores R_2_Ti_2_O_7_ (R = rare earth) by the optical floating-zone method. J. Cryst. Growth.

[2] Yu J (2004). Giant dielectric constant of hexagonal BaTiO_3_ crystal grown by containerless processing. Chem. Mater..

[3] Wechsler BA, Klein MB, Rytz D (1987). Crystal growth, processing, and characterization of photorefractive BaTiO_3_. Proc. SPIE Int. Soc. Opt. Eng..

[4] Nakamura T (2005). Preferential formation of anatase in laser-ablated titanium dioxide films. Acta Mater..

[5] Yao S, Liu T, Li C, Yang G, Li C (2017). Epitaxial growth during the rapid solidification of plasma-sprayed molten TiO_2_ splat. Acta Mater..

[6] Zheng K, Zhang Z, Liu L, Wang X (2014). Investigation of the viscosity and structural properties of CaO-SiO_2_-TiO_2_ slags. Metall. Mater. Trans. B.

[7] Park H, Park J, Kim GH, Sohn I (2012). Effect of TiO_2_ on the viscosity and slag structure in blast furnace type slags. Steel Res. Int..

[8] Mao Z, Wang C, Zheng X, Yu J, Xiong L (2018). Optical properties, thermal stability, and forming region of high refractive index La_2_O_3_-TiO_2_-Nb_2_O_5_ glasses. J. Am. Ceram. Soc..

[9] Masuno A (2012). Glass-forming region and high refractive index of TiO2-based glasses prepared by containerless processing. Phys. Status Solidi C.

[10] Li, Ishigaki T (2001). Synthesis of crystalline micron spheres of titanium dioxide by thermal plasma oxidation of titanium carbide. Chem. Mater..

[11] Yu J (2005). Synthesis of barium titanate by electrostatic levitation. J. Cryst. Growth.

[12] Yu JD, Paradis PF, Ishikawa T, Yoda S (2004). Microstructure and dielectric constant of BaTiO_3_ synthesized by roller quenching. Jpn. J. Appl. Phys. Pt 1.

[13] Vekilov PG (2010). Nucleation. Cryst. Growth Des..

[14] Nielsen MH, Aloni S, De Yoreo JJ (2014). In situ TEM imaging of CaCO_3_ nucleation reveals coexistence of direct and indirect pathways. Science.

[15] Desgranges C, Delhommelle J (2007). Controlling polymorphism during the crystallization of an atomic fluid. Phys. Rev. Lett..

[16] Tang X (2017). Local structure order assisted two-step crystal nucleation in polyethylene. Phys. Rev. Mater..

[17] An S (2018). Common mechanism for controlling polymorph selection during crystallization in supercooled metallic liquids. Acta Mater..

[18] Hamasaki Y (2016). Crystal isomers of ScFeO_3_. Cryst. Growth Des..

[19] Ostwald WZ (1897). Studies on formation and transformation of solid materials. Z. Phys. Chem..

[20] Ishihara KN, Maeda M, Shingu PH (1985). The nucleation of metastable phases from undercooled liquids. Acta Metall..

[21] Kuribayashi K, Kumar MSV (2011). Metastable phase formation from undercooled melt of oxide material. J. Phys. Conf. Ser..

[22] Ten WP, Ruiz-Montero MJ, Frenkel D (1995). Numerical evidence for bcc ordering at the surface of a critical fcc nucleus. Phys. Rev. Lett..

[23] Zhang TH, Liu XY (2009). Nucleation: what happens at the initial stage?. Angew. Chem. Int. Ed. Engl..

[24] Desgranges C, Delhommelle J (2007). Molecular insight into the pathway to crystallization of aluminum. J. Am. Chem. Soc..

[25] Myerson AS, Trout BL (2013). Nucleation from solution. Science.

[26] An, S. et al. Two-step crystal growth mechanism during crystallization of an undercooled Ni_50_Al_50_ alloy. *Sci. Rep.* **6** (2016). 10.1038/srep31062 PMC4971477 27486073

[27] Alexander S, McTague J (1978). Should all crystals be bcc? Landau theory of solidification and crystal nucleation. Phys. Rev. Lett..

[28] Li J, Wang Z, Deepak FL (2018). Direct Atomic-scale observation of intermediate pathways of melting and crystallization in supported Bi nanoparticles. J. Phys. Chem. Lett..

[29] Zhou J (2019). Observing crystal nucleation in four dimensions using atomic electron tomography. Nature.

[30] McCormack SJ, Weber RJ, Kriven WM (2018). In-situ investigation of Hf_6_Ta_2_O_17_ anisotropic thermal expansion and topotactic, peritectic transformation. Acta Mater..

[31] Benmore CJ, Weber JKR (2017). Aerodynamic levitation, supercooled liquids and glass formation. Adv. Phys. X.

[32] Skinner LB (2014). Low cation coordination in oxide melts. Phys. Rev. Lett..

[33] Alderman OLG, Benmore, Lin A, Tamalonis A, Weber JKR (2018). Borate melt structure: Temperature-dependent B-O bond lengths and coordination numbers from high-energy X-ray diffraction. J. Am. Ceram. Soc..

[34] Kohara S (2014). Atomic and electronic structures of an extremely fragile liquid. Nat. Commun..

[35] Gallo P (2016). Water: a tale of two liquids. Chem. Rev..

[36] Akola J (2013). Network topology for the formation of solvated electrons in binary CaO-Al_2_O_3_ composition glasses. Proc. Natl Acad. Sci. USA.

[37] Matsumura S, Watanabe M, Mizuno A, Kohara S (2007). Supercooled barium boric oxide melts: X-ray diffraction measurements and glass formation. J. Am. Ceram. Soc..

[38] Ansell S (1997). Structure of liquid aluminum oxide. Phys. Rev. Lett..

[39] Ge X (2019). Polymorphic transition and nucleation pathway of barium dititanate (BaTi_2_O_5_) during crystallization from undercooled liquid. Sci. Rep..

[40] Dawson JA, Freeman CL, Ben LB, Hardening JH, Sinclair DC (2011). An atomistic study into the defect chemistry of hexagonal barium titanate. J. Appl. Phys..

[41] Ebralidze I, Lyahovitskaya V, Zon I, Wachtel E, Lubomirsky I (2005). Anomalous pre-nucleation volume expansion of amorphous BaTiO_3_. J. Mater. Chem..

[42] Wachtel E, Lubomirsky I (2010). Quasi-amorphous inorganic thin films: non-crystalline polar phases. Adv. Mater..

[43] Nagashio K, Kuribayashi K (2002). Phase selection in the undercooled peritecticy Y_3_Fe_5_O_12_ melt. Acta Mater..

[44] Wu YH (2017). A triple comparative study of primary dendrite growth and peritectic solidification mechanism for undercooled liquid Fe_59_ Ti_41_ alloy. Acta Mater..

[45] Lee K (2012). Thermophysical properties of BaTiO_3_ ceramics prepared by aerodynamic levitation. Thermochim. Acta.

[46] Kirby KW, Wechsler BA (1991). Phase relations in the barium titanate-titanium oxide system. J. Am. Ceram. Soc..

[47] Alderman OLG (2016). Continuous structural transition in glass-forming molten titanate BaTi_2_O_5_. J. Phys. Chem. C.

[48] Wilding MC, Wilson M, Benmore CJ, Weber JKR, McMillan PF (2013). Structural changes in supercooled Al_2_O_3_-Y_2_O_3_ liquids. Phys. Chem. Chem. Phys..

[49] Greaves GN (2008). Detection of first-order liquid/liquid phase transitions in yttrium oxide-aluminum oxide melts. Science.

[50] Doolittle AK (1951). Studies in newtonian flow. II. The dependence of the viscosity of liquids on free-space. J. Appl. Phys..

[51] Shannon RD, Prewitt CT (1969). Effective ionic radii in oxides and fluorides. Acta Crystallogr. B.

[52] Alderman OLG (2016). Temperature-driven structural transitions in molten sodium borates Na_2_O-B_2_O_3_: X-ray diffraction, thermodynamic modeling, and implications for topological constraint theory. J. Phys. Chem. C.

[53] Skinner LB (2013). A time resolved high energy X-ray diffraction study of cooling liquid SiO_2_. Phys. Chem. Chem. Phys..

[54] Alderman OLG (2015). Liquid B_2_O_3_ up to 1700 K: X-ray diffraction and boroxol ring dissolution. J. Phys. Condens. Matter.

[55] Alderman OLG, Skinner LB, Benmore CJ, Tamalonis A, Weber JKR (2014). Structure of molten titanium dioxide. Phys. Rev. B..

[56] Brown ID, Altermatt D (1985). Bond-valence parameters obtained from a systematic analysis of the inorganic crystal structure database. Acta Crystallogr. B.

[57] Hennet L (2007). Development of structural order during supercooling of a fragile oxide melt. J. Chem. Phys..

[58] Russo, J. & Tanaka, H. The microscopic pathway to crystallization in supercooled liquids. *Sci. Rep.* **2**, 505 (2012). 10.1038/srep00505 PMC3395031 22792437

[59] Kelton KF (2003). First x-ray scattering studies on electrostatically levitated metallic liquids: demonstrated influence of local icosahedral order on the nucleation barrier. Phys. Rev. Lett..

[60] Weber JK, Krishnan S, Ansell S, Hixson AD, Nordine PC (2000). Structure of liquid Y_3_Al_5_O_12_ (YAG). Phys. Rev. Lett..

[61] Nakatani T (2016). Variable-temperature single-crystal X-ray diffraction study of tetragonal and cubic perovskite-type barium titanate phases. Acta Crystallogr. B.

[62] Akimoto J, Gotoh Y, Oosawa Y (1994). Refinement of hexagonal BaTiO_3_. Acta Crystallogr. C.

[63] Zhang H, Li N, Li K, Xue D (2007). Structural stability and formability of ABO_3_-type perovskite compounds. Acta Crystallogr. B.

[64] Kumar MV, Kuribayashi K, Yu J, Okada JT, Ishikawa T (2013). Microstructure and magnetic properties of metastable RFeO_3_ (R: Rare-earth element) formed from undercooled melt. J. Am. Ceram. Soc..

[65] Vijaya Kumar MS, Higaki N, Kuribayashi K, Hibiya T, Yoda S (2011). Formation of orthorhombic and multiferroic hexagonal phases from an undercooled RMnO_3_ (R = Rare-Earth Element) melt using a containerless technique. J. Am. Ceram. Soc..

[66] Lin MH, Lu HY (2001). Hexagonal-phase retention in pressureless-sintered barium titanate. Philos. Mag. A.

[67] Wang Y (2014). Atomistic insight into viscosity and density of silicate melts under pressure. Nat. Commun..

[68] Krishnan S (1997). Levitation apparatus for structural studies of high temperature liquids using synchrotron radiation. Rev. Sci. Instrum..

[69] Lorch E (1969). Neutron diffraction by germania, silica and radiation-damaged silica glasses. J. Phys. C Solid State Phys..

[70] Langstaff D, Gunn M, Greaves GN, Marsing A, Kargl F (2013). Aerodynamic levitator furnace for measuring thermophysical properties of refractory liquids. Rev. Sci. Instrum..

[71] Kresse G, Furthmüller J (1996). Efficiency of ab-initio total energy calculations for metals and semiconductors using a plane-wave basis set. Comput. Mater. Sci..

[72] Perdew JP, Wang Y (1992). Accurate and simple analytic representation of the electron-gas correlation energy. Phys. Rev. B Condens. Matter.

[73] Blöchl PE (1994). Projector augmented-wave method. Phys. Rev. B.

[74] Kresse G, Joubert D (1999). From ultrasoft pseudopotentials to the projector augmented-wave method. Phys. Rev. B..

[75] Perdew JP, Burke K, Ernzerhof M (1996). Generalized gradient approximation made simple. Phys. Rev. Lett..

[76] Nosé S (1984). A unified formulation of the constant temperature molecular dynamics methods. J. Chem. Phys..

[77] Alderman OLG, Benmore CJ, Neuefeind J, Tamalonis A, Weber R (2019). Molten barium titanate: a high-pressure liquid silicate analogue.. J. Phys. Condens. Matter.

