# Supplementary material for: Anomalous structure transition in undercooled melt regulates polymorphic selection in barium titanate crystallization
Source: Commun Chem. 2021 Mar 2;4:27. doi: 10.1038/s42004-021-00462-w (PMC9814643; doi:10.1038/s42004-021-00462-w)
Supplement: Supplementary file 1 — Supplementary Information [file 42004_2021_462_MOESM1_ESM.pdf]

Supplementary Information:

Anomalous structure transition in undercooled melt regulates polymorphic selection in barium titanate crystallization

Xuan Ge[a], Qiaodan Hu[a*], Fan Yang[b], Jun Xu[a], Yanfeng Han[a], Pingsheng Lai[a], Jingyu Qin[c], Jianguo Li[a]

[a] *Shanghai Key Laboratory of Materials Laser Processing and Modification, School of Materials Science and Engineering, Shanghai Jiao Tong University, 200240 Shanghai, P. R. China*

[b] *School of Mechanical Engineering, Shanghai Jiao Tong University, 200240 Shanghai, P. R. China*

[c] *School of Materials Science and Engineering, Shandong University, 250100 Jinan, P. R. China*

Corresponding author: Prof., Ph.D., Qiaodan Hu, E-mail address: qdhu@sjtu.edu.cn

Supplementary Note 1. Microstructure and phase compositions

Nucleation was triggered at a series of undercoolings and a multi-path crystallization behavior was observed. If triggered nucleation at small undercoolings (ΔT = 78.1 K and 177.9 K), both *h*-BT and *t*-BT were detected after solidification. On the contrary, if triggered nucleation at large undercoolings, only *t*-BT can be obtained. Analogous results were also reported by Yu *et al*[1].

To clarify the origin of the polymorphic selection behavior of BT, microstructural analyses were carried out. Fig. S1(a)-(c) present the SEM images of three selected regions (top, middle and bottom) of a solidified sample. As shown in Fig. S1(a), a layer of fine equiaxed grains with a thickness of ~ 50 μm is observed near the top boundary of the sample. Below this boundary layer, grains become obviously large, and solidification defects (cracks or shrinkage cavity) are more commonly observed. The middle region of the sample mainly consists of coarse equiaxed crystals, as shown in Fig. S1(b). Near the triggering point at the bottom, columnar-like BT crystals are formed (Fig. S1(c)). These crystals are aligned in parallel with growth direction perpendicular to the sample boundary.

Formation of the above microstructure is illustrated by a schematic diagram (Fig. S1(d)). At the quenching spot (bottom), the droplet contacts with the water-cooled nozzle directly. Heat is conducted rapidly from the droplet to the nozzle in a direction perpendicular to the contact surface, which results in a preferential crystal growth along the direction of heat flow. On the top of the droplet, a local quenching zone appears due to the fastest cooling rate, which results from an uneven temperature distribution within the suspending droplet[2]. In this region, homogeneous nucleation and no-directional heat flow result in formation of fine equiaxial crystals. The middle part of the sample is the final solidified region due to release of latent heat of crystallization and the lowest heat transport rate. Low nucleation rate facilitates the emergence of coarse equiaxial crystals.

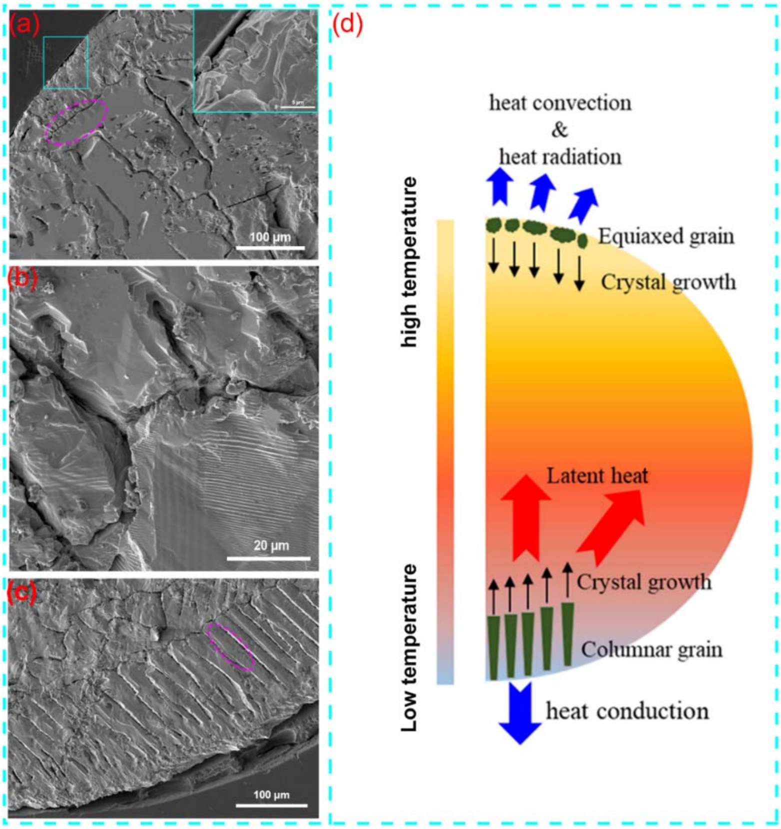

Fig S1. **Cross-sectional SEM images of three selected regions of a solidified BT sphere triggered nucleation at a small undercooling (ΔT = 71 K)**. (a)-(c) top, middle and bottom region, respectively. Inset figure in (a) is the expanded view of the area in the rectangular frame, showing the fine equiaxed crystals near the outer edge of the sample. (d) Schematic illustration of heat transport and crystal growth in molten BT after triggering nucleation on the wall of nozzle.

To identify the distribution of hexagonal and tetragonal phases, Raman mapping analysis is employed. *h*-BT and *t*-BT have distinct characteristic Raman peaks[3,4], as shown in Fig. S2(a). The regional distribution of two phases in the above-mentioned three typical regions is presented in Fig. S2(b)-(d). At the top side of the sample, only *h*-BT is detected; on the contrary, only *t*-BT presents near the vicinity of the triggering point. In the middle of the sample, a hybrid phase distribution is observed, which has a distinct dividing line along the grain boundary. Combining the microstructure features and the regional distribution of two phases, we concluded that: i) both *c*-BT and *h*-BT nucleate from melt directly, rather than from a solid-state phase transition; ii) the undercooling before crystal nucleation of *c*-BT is larger than that of *h*-BT because the quenching effect of the water-cooled nozzle (triggering point) is much stronger than natural cooling (the top side). iii) The possibility of cross-nucleation in *c*-/*h*-BT selection is low, although it's very common in simpler condensed matter systems[5,6], as these two phases present obvious regional distribution rather than coupled distribution.

Polymorphic selection in undercooled melt during solidification is often accompanied by a change in crystal morphology, as observed in $RMnO_3$ (R = rare-earth element)[7] and our previous work[8], which indicates different crystal growth modes of different phases. However, such effect is not observed in BT. Fig. S3(a) and (b) show the high-magnification images of two phases highlighted by magenta ellipses in Fig. S1(a) and (c), respectively. Obvious growth steps can be observed on the edge of the grain boundary, which suggests that the crystal growth of both phases presents typical facet mode.

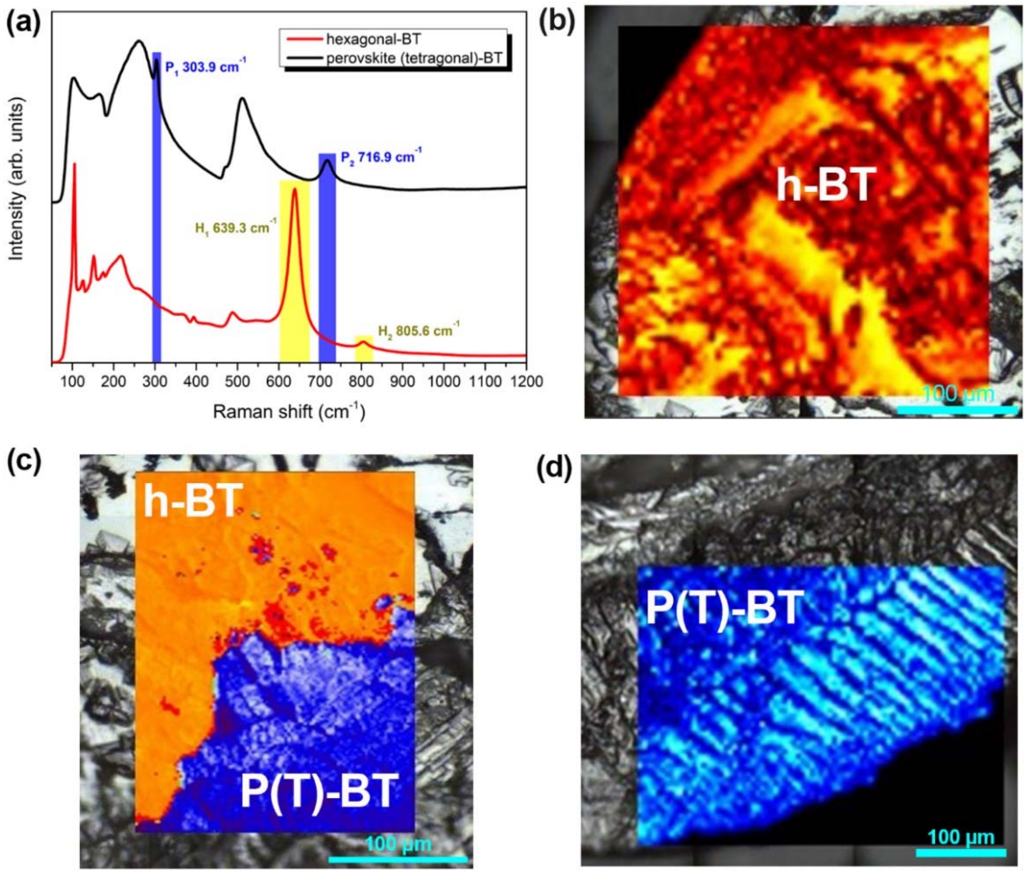

Fig. S2. **Raman results of BT polymorphic selection.** (a) Raman spectra of *h*-BT and *t*-BT (perovskite). Characteristic Raman peaks at 639.3 cm^{-1} and 805.6 cm^{-1} of *h*-BT are highlighted in yellow and those at 303.9 cm^{-1} and 716.9 cm^{-1} for *t*-BT are highlighted in blue. (b)-(d) Raman imaging of the regions shown in Fig. S1 (a)-(c). Distinction between *h*- and *t*-BT is illustrated by integrating the characteristic Raman peak for each phase (hexagonal: 639.3 cm^{-1}; tetragonal: 716.9 cm^{-1}.

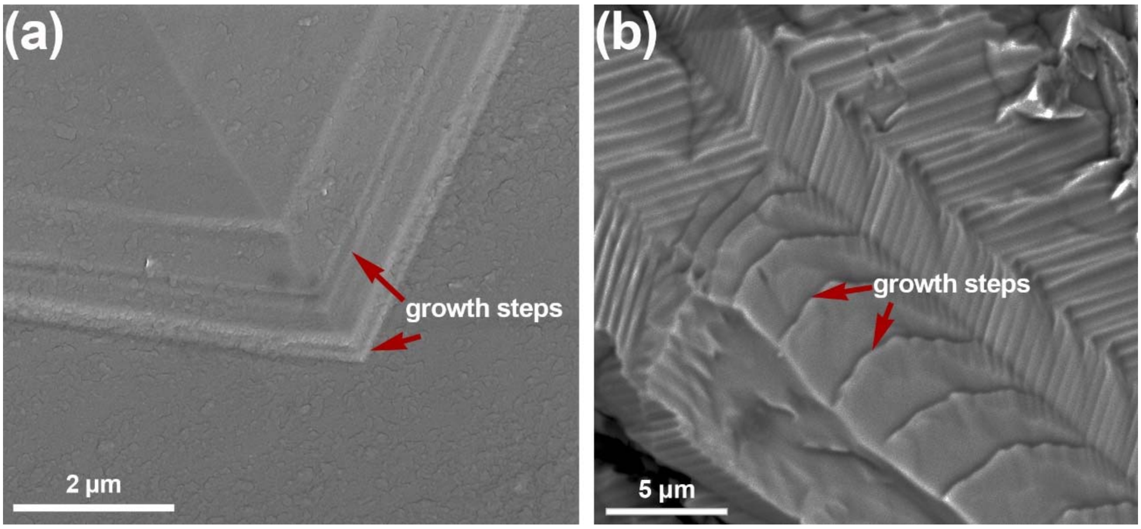

Fig. S3. **High-resolution SEM results.** (a) and (b) represents regions marked by magenta ellipses in Fig. S1(a) and (c) respectively, showing the steps for crystal growth.

Supplementary Note 2. Two-dimensional diffraction patterns

Two representative solidification processes were tracked by real time HEXRD experiments. X-ray photon beam is incident on the top-side of the sample (Fig. S4) and their two-dimensional diffraction patterns were recorded during the crystallization process.

1) Large undercooling ($\Delta T = 644$ K). Two-dimensional diffraction patterns recorded in crystallizing from supercooled liquid ($\Delta T = 644$ K) are illustrated as a function of time (Fig. S5). The undercooled melt presents as diffuse scattering rings ($t1$ and $t2$). After recalescence, a few diffraction spots appear ($t3$), indicating that the primary *c*-BT phase nucleates from the undercooled liquid. These spots are weak and scarce, suggesting that the primary *c*-BT is very limited in volume and number, which confirms that crystallization in undercooled melt often commences with only a few nuclei. Over time, more concentric polycrystalline diffraction rings are detected ($t4$, $t7$ and $t10$) corresponding to new nucleation or grain growth during crystallization. These concentric diffraction rings remain stable over time without occurrence of new diffraction spots, suggesting absence of any secondary phase or solid-state phase transition during the crystallization process of supercooled BT melt.

2) Small undercooling ($\Delta T = 71$ K). Fig. S6 presents the two-dimensional diffraction patterns recorded for BT that triggered nucleation at a low undercooling ($\Delta T = 71$ K). The integral result of each diffraction pattern is given as inset figure. Before triggering, the diffuse scattering rings illustrate the melt feature ($t1$ and t2). After triggering, *c*-BT crystals grow rapidly from the triggering point and the corresponding sharp diffraction spots appear immediately ($t3$). Diffuse scattering rings coexist with diffraction spots, indicating that liquid phase still exists in the exposure region (top side of sample). At $t4$, characteristic diffraction spots of *h*-BT are detected and the diffraction rings of *c*-BT remain unchanged, suggesting that *h*-BT nucleates directly from the residual liquid. At $t5$, intensity of the diffraction rings is reduced greatly and the feature of the melt is clear, which correspond to re-melting in the diffraction region due to release of latent heat. In

the late stage of solidification ($t6$, $t8$ and $t10$), more distinct diffraction spots are observed illustrating that the number of grains is reduced but the size is expanded during the re-melting stage, which is consistent with the microstructure shown in Fig. S1(b). Furthermore, comparing the integral diffraction patterns before re-melting ($t3$ and $t4$) with those after re-melting ($t6$-$t12$), it is found that the patterns resemble more to *h*-BT after re-melting, which demonstrates that part of *c*-BT disappears due to its lower thermal stability at the re-melting temperature (near melting point).

It should be pointed out that the collection time is much longer than the time scale of nucleation, but it is sufficient to determine whether the solid-state phase transition occurs during the crystallization process[9]. The real time HEXRD experiment proves that both *h*-BT and *c*-BT (perovskite) nucleate directly from undercooled melt. *c*-BT precipitates from the triggering point where the undercooling is large, and *h*-BT nucleates from the melt at the top side of the droplet where the undercooling is low. Therefore, coexistence of two phases within the sample triggered nucleation at a small undercooling is attributed to uneven temperature distribution and nucleation sequences manipulated by undercoolings.

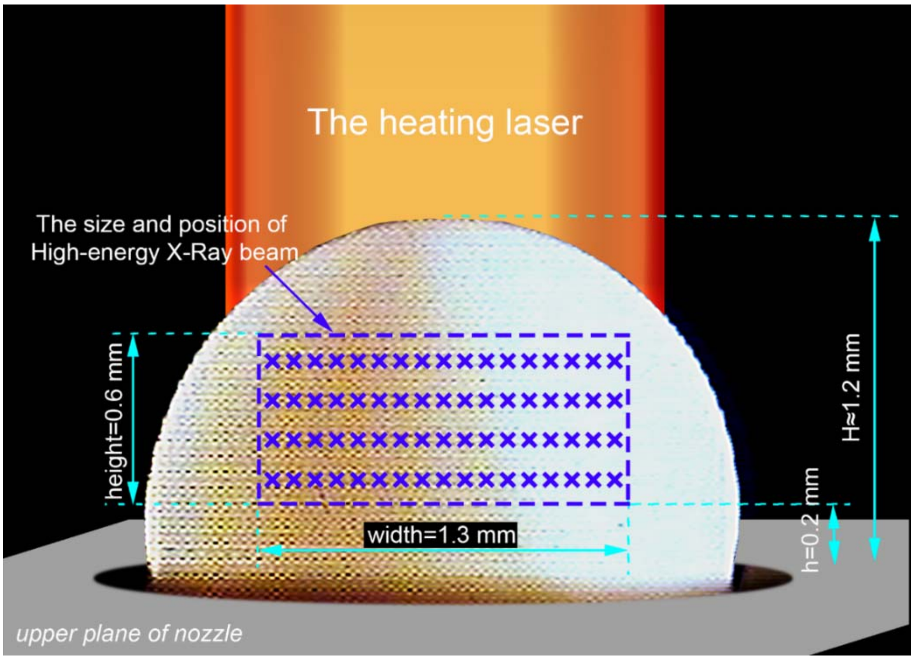

Fig. S4. Magnified optical image of the levitated droplet and geometric parameters depicting the high-energy X-ray beam size and position.

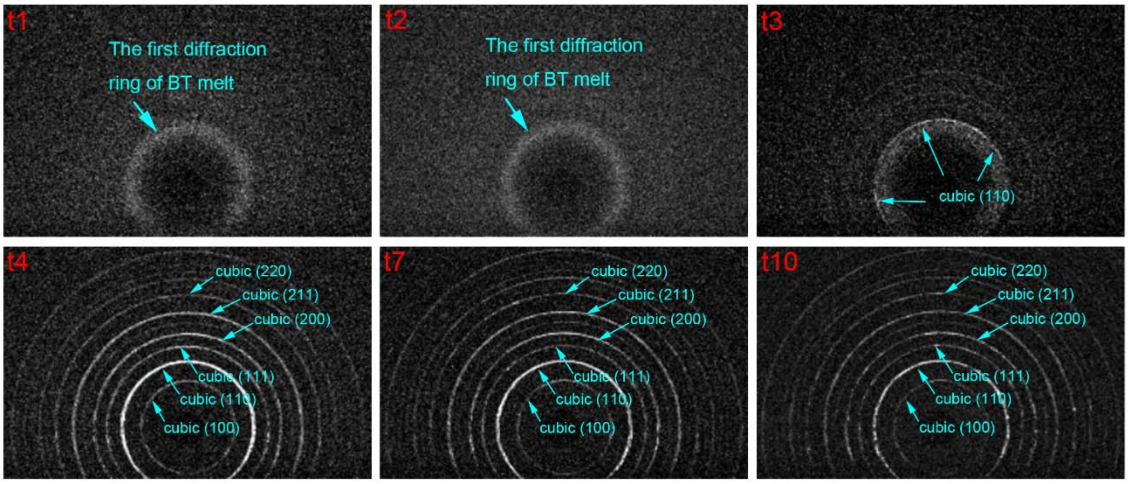

Fig. S5. **Two-dimensional diffraction patterns of BT triggered crystallization at a large undercooling (Δ*T* = 644 K).** Images were captured by a PE panel with 0.5 s exposure time. The patterns are stacked in sequence of elapsing time (*t*5-*t*6 and *t*8-*t*9 are not shown here because they are identical to t4, t7 and t10).

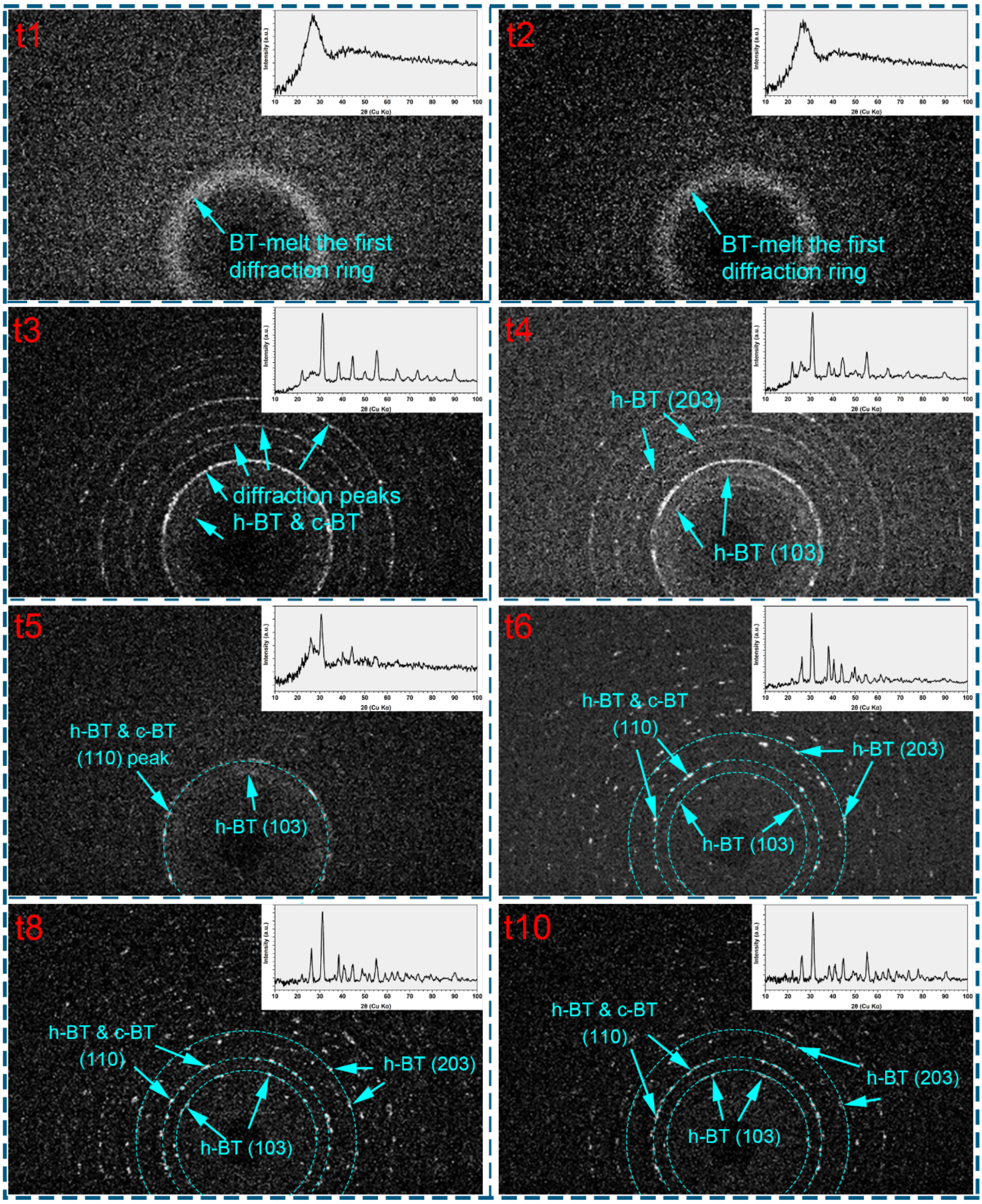

Fig. S6. Two-dimensional diffraction patterns of BT triggered crystallization at a low undercooling (ΔT = 71 K). The top right inset figure in each diffraction pattern shows the integrated result using Fit2D software. Some patterns (*t*7, *t*11 and *t*12) are not list here as they are the same as *t*6, *t*8 and *t*10.

Supplementary Note 3. Density of molten BT

Density of molten BT has been previously measured by Paradis *et al.* under a high pressure (450 kPa) in N_2 based on pressurized electrostatic levitation (PESL). They reported a good linear relationship between ρ and T: $\rho_L^{BT} = 4.04 - 3.4 \times 10^{-4} T$ (in Kelvin and grams per centimeter) in the temperature range between 1300 and 2025 K[10]. However, the experimental conditions of Paradis are not in accordance with those of our HEXRD experiment. Density of molten oxides is quite sensitive to pressure and atmosphere, for example, Kumar *et al.* measured the density of molten $CaAl_2O_4$ (CA) at various atmospheres (oxygen, air and nitrogen) and found that the density of CA melt decreased with increasing oxygen partial pressure[11].Therefore, we re-measure the density of molten BT under our experimental condition. A schematic view of the experimental setup is given in Fig S7.

For density measurement, the temperature-time profile was recorded, Fig S8(a). Real-time dimensional changes of the molten drops were captured by a high-speed camera. The imaging acquisition frequency was set five times of the temperature recording frequency (50 per second). Therefore, five density values were obtained for each temperature. Average value and the associated error bar at a certain temperature are calculated from these five results.

The measured densities at various temperatures are presented in Fig. S8(b). Data from Paradis *et al.* are also included for comparison. It can be seen that the density values from this work and those from Ref.10 are quite close, but the ρ-T relationship is very different. Under the pressurized nitrogen atmosphere condition, the melt density of BT exhibits a good linear relationship with temperature from superheating to undercooling state. In contrast, under ambient pressure and oxygen atmosphere (experimental condition in our work), ρ-T deviates from linear relationship and can be better described by a piecewise relation:

$$\begin{cases} \rho_L = 4.299 - 1.96 \times 10^{-4} T \quad (T_m < T \leq 2050 \text{ K}) \\ \rho_L = 5.431 - 8.21 \times 10^{-4} T \quad (1300 \text{ K} < T \leq T_m) \end{cases}$$

The change of ρ-T relationship indicates that BT melt may go through a distinct structural or dynamic evolution when quenching from superheating state to undercooling state. Extrapolating the density of molten BT to room temperature yields a value of ~ 5.21 g cm^{-3}, which is very close to the density of BT amorphous film fabricated by radio-frequency plasma sputtering[12].

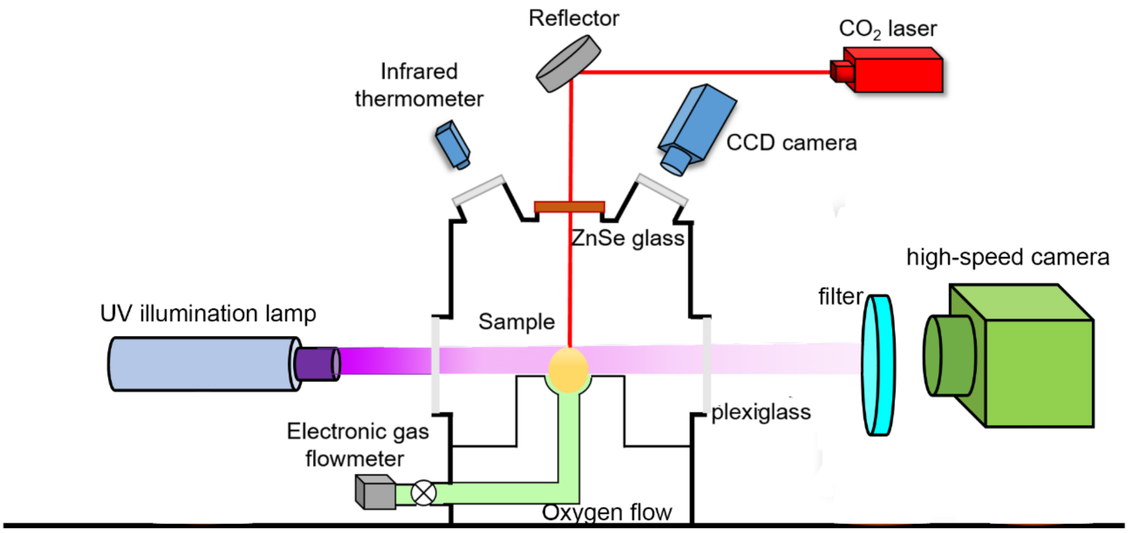

Fig. S7. Schematic view of the facility used for density measurement.

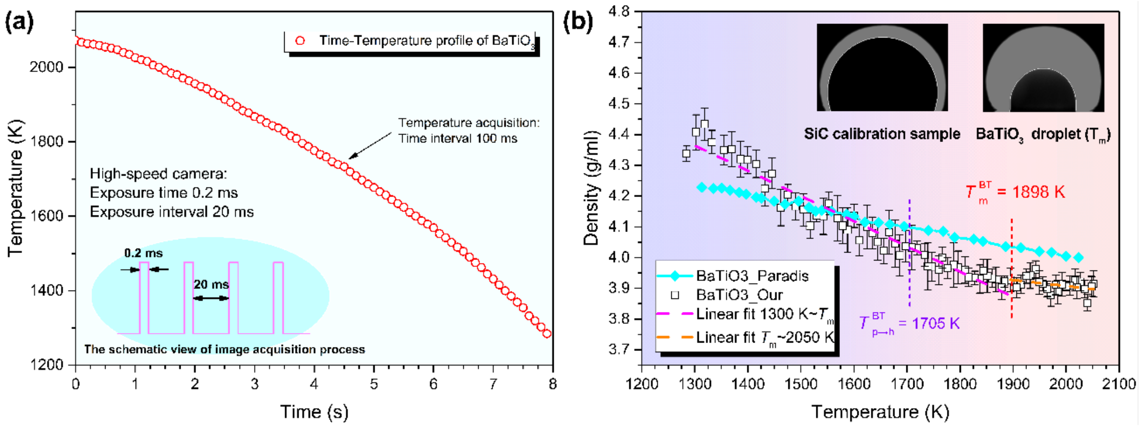

Fig. S8. **Schemes and results of density measurement.** (a) The temperature-time profile of continuous cooling process. The image acquisition frequency of high-speed camera is also shown in the figure. (b) Density of BT melt as a function of temperature. Data from Paradis *et al.*[10] are included for comparison. The top-right inset figures are the UV backlighted imaging of a SiC bead for calibration (left) and the molten BT droplet (right).

Supplementary Note 4. Pair weighting factors W_{ij}

The pair weighting factor W_{ij}, showing the contribution to the total X-ray scattering intensity of each atomic pair in BT, is calculated using concentration of atoms c_i and X-ray atomic form factor of elements $f_i(Q)$ according to:

$$W_{ij}(Q) = 2c_i c_j f_i(Q) f_j(Q) / (\sum_i^n c_i f_i(Q))^2$$

The calculated W_{ij} of six atomic pairs in BT is plotted as a function of wave vector Q, as shown in Fig. S9. Average value of $W_{Ti\text{-}O}$ in the Q range from 0 to Q_{lim} (~ 4.33 Å^{-1}) is used to calculate the Ti-O coordination number, in which Q_{lim} is obtained by $Q = {}^{2\pi}/_{r}$ using the minimum value of r (~1.45 Å) of the Ti-O peak.

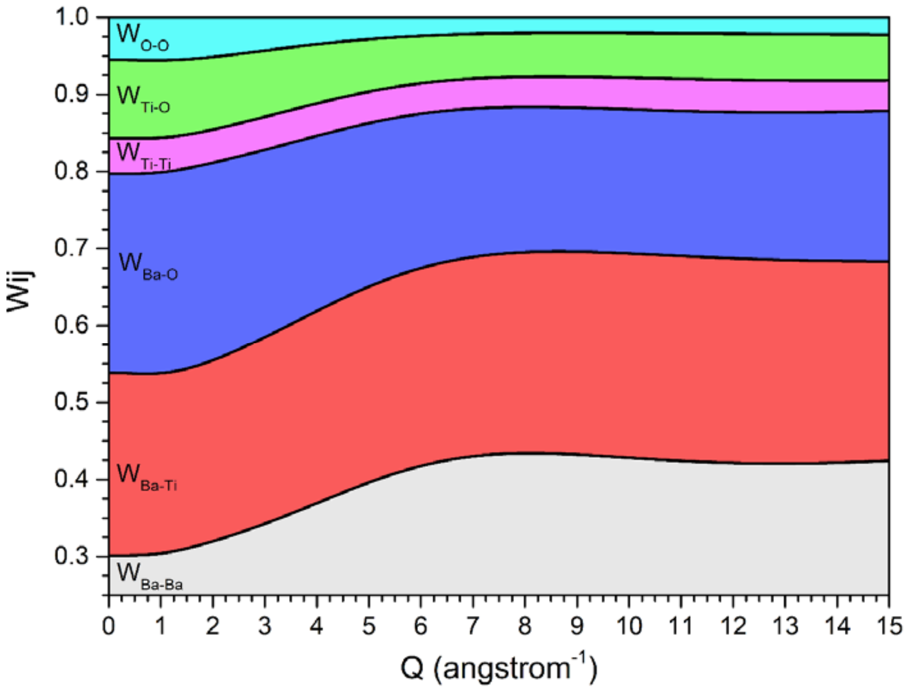

Fig. S9. The Q dependence of weighting factors of six atomic pairs in BT

Supplementary Note 5. Verifying the robust of the structural transition determined by AIMD

Differing from the initial AIMD simulation run, a re-run parallel AIMD simulation was performed from different starting configurations. The total simulation time of re-run

AIMD simulation was set 20 ps with timesteps of 2 fs, and the last 10 ps configurations was used to calculate the Ti-O coordination number (CN_{TiO}). As shown in Fig. S10(a), the CN_{TiO} results are slightly different from the initial AIMD running, but the overall trend are not changed. Most important is that the discontinuous CN_{TiO} transition taking place between 1583 K and 1523 K is still robust, which is accordant with our finding and conclusion in manuscript. We must admit that both the new starting configuration and the initial starting configuration are generated by fitting our experimental structural functions through running Reverse Monte Carlo (RMC) simulation, and the total energy of two ensembles are quite approximate.

In order to further test the anomalous CN_{TiO} transition, we construct a random starting configuration, in which we only ensure the atoms are not too close to each other (matching the cutoff radius) without fitting the experimental correlation functions. This random starting configuration was first relaxed at 3000 K for over 10 ps, and then quenched to transition temperatures (1583 K and 1523 K) for another 20 ps equilibration. As presented in Fig. S10(b), the random starting configurations correspond to a higher total energy even after AIMD simulation, which indicates that the ensembles originating from the random starting configurations are relatively metastable state compared with that from the initial starting configurations. We compared the CN_{TiO} and found the transition was still existed, but the magnitude of CN_{TiO} transition is just half of that determined by the initial starting configuration.

In summary, we believe and convince that the discontinuously anomalous structure transition exists in undercooled BT liquid. The conclusion is robust and can be reproduced by diffraction experiments as well as AIMD simulation. One important thing should be emphasized that the transition magnitude determined by AIMD may be closely related to the total energy state of starting configuration. A relatively stable (lower total energy) starting liquid configuration may be more helpful for studying this liquid-liquid structural transition.

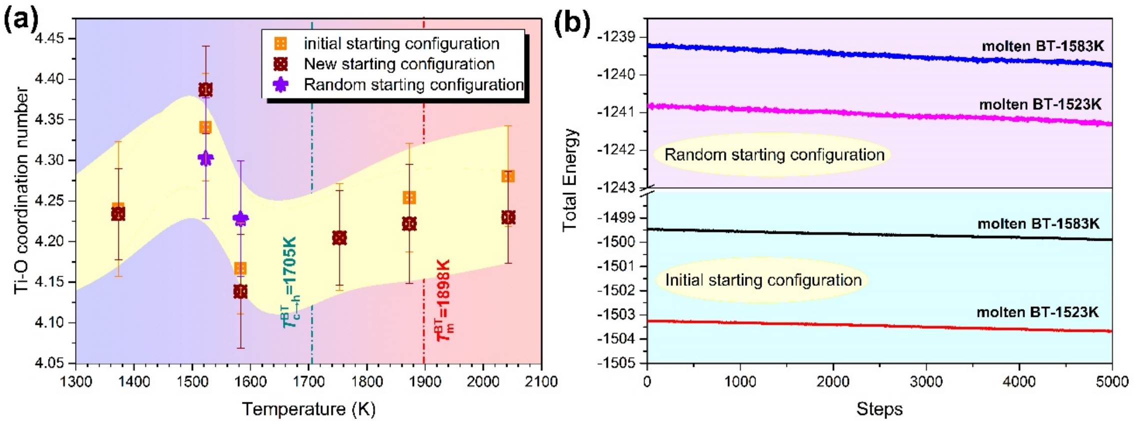

Fig. S10. Identifying structure transition in undercooling BT melt using different starting configuration in AIMD simulation. (a) The variation of CN_{TiO} determined by running AIMD from different starting configurations. (b) the total energy comparison of ensembles that stem from the initial starting configuration and random staring configuration, respectively.

Supplementary References:

1. Yu, J. et al. Giant Dielectric Constant of Hexagonal $BaTiO_3$ Crystal Grown by Containerless Processing. *Chem Mater* **16**, 3973-3975 (2004).
2. Ge, X. et al. Ambiguous temperature difference in aerodynamic levitation process: Modelling, solving and application. *J Mater Sci Technol* **35**, 1636-1643 (2019).
3. Yamaguchi, H., Uwe, H., Sakudo, T. & Sawaguchi, E. Raman-Scattering Study of the Soft Phonon Modes in Hexagonal Barium Titanate. *J Phys Soc Jpn* **56**, 589-595 (1987).
4. Hayashi, H., Nakamura, T. & Ebina, T. In-situ Raman spectroscopy of $BaTiO_3$ particles for tetragonal - cubic transformation. *J Phys Chem Solids* **74**, 957-962 (2013).
5. Desgranges, C. & Delhommelle, J. Controlling polymorphism during the crystallization of an atomic fluid. *Phys Rev Lett* **98**, 235502 (2007).
6. An, S. et al. Common mechanism for controlling polymorph selection during crystallization in supercooled metallic liquids. *Acta Mater* **161**, 367-373 (2018).
7. Vijaya Kumar, M.S., Higaki, N., Kuribayashi, K., Hibiya, T. & Yoda, S. Formation of Orthorhombic and Multiferroic Hexagonal Phases from an Undercooled $RMnO_3$ (R=Rare-Earth Element) Melt Using

a Containerless Technique. *J Am Ceram Soc* **94**, 281-288 (2011).

8. Ge, X. et al. Polymorphic transition and nucleation pathway of barium dititanate ($BaTi_2O_5$) during crystallization from undercooled liquid. *Sci Rep-UK* **9**, 7207 (2019).
9. Wang, Y. et al. Metastable solidification of hypereutectic Co_2Si-CoSi composition: Microstructural studies and in-situ observations. *Acta Mater* **142**, 172-180 (2018).
10. Paradis, P.F., Yu, J., Ishikawa, T., Aoyama, T. & Yoda, S. Contactless density measurement of high-temperature $BiFeO_3$ and $BaTiO_3$. *Applied Physics A* **79**, 1965-1969 (2004).
11. Kumar, M.V., Okada, J.T., Ishikawa, T., Paradis, P. & Watanabe, Y. Density measurement of glass and liquid $CaAl_2O_4$ using a pressurized electrostatic levitator. *Meas Sci Technol* **25**, 085301 (2014).
12. Ebralidze, I., Lyahovitskaya, V., Zon, I., Wachtel, E. & Lubomirsky, I. Anomalous pre-nucleation volume expansion of amorphous $BaTiO_3$. *Journal of Materials Chemistry* **15**, 4258 (2005).
